# Anticancer Therapies Based on Oxidative Damage: *Lycium barbarum* Inhibits the Proliferation of MCF-7 Cells by Activating Pyroptosis through Endoplasmic Reticulum Stress

**DOI:** 10.3390/antiox13060708

**Published:** 2024-06-11

**Authors:** Maria Rosaria Miranda, Manuela Giovanna Basilicata, Vincenzo Vestuto, Giovanna Aquino, Pasquale Marino, Emanuela Salviati, Tania Ciaglia, Gloria Domínguez-Rodríguez, Ornella Moltedo, Pietro Campiglia, Giacomo Pepe, Michele Manfra

**Affiliations:** 1 Department of Pharmacy, University of Salerno, Via G. Paolo II, 84084 Fisciano, Italy; mmiranda@unisa.it (M.R.M.); gaquino@unisa.it (G.A.); esalviati@unisa.it (E.S.); tciaglia@unisa.it (T.C.); pcampiglia@unisa.it (P.C.); 2 PhD Program in Drug Discovery and Development, University of Salerno, 84084 Fisciano, Italy; moltedo@unisa.it; 3 Department of Advanced Medical and Surgical Sciences, University of Campania “Luigi Vanvitelli”, 80138 Naples, Italy; manuelagiovanna.basilicata@unicampania.it; 4 Department of Science, University of Basilicata, Viale dell’Ateneo Lucano 10, 85100 Potenza, Italy; pasqualemarino.pm90@gmail.com (P.M.); michele.manfra@unibas.it (M.M.); 5 Departamento de Química Analítica, Química Física e Ingeniería Química, Facultad de Ciencias, Universidad de Alcalá, Ctra. Madrid-Barcelona Km. 33.600, 28871 Alcalá de Henares, Madrid, Spain; gloria.dominguezr@uah.es; 6 National Biodiversity Future Center (NBFC), 90133 Palermo, Italy

**Keywords:** goji berries, MCF-7, ER stress, pyroptosis, onconutraceutical, pro-oxidants

## Abstract

*Lycium barbarum*, commonly recognized as goji berry or wolfberry, is highly appreciated not only for its organoleptic and nutritional properties but also as an important source of bioactive compounds such as polysaccharides, carotenoids, phenolics, and various other non-nutritive compounds. These constituents give it a multitude of health benefits, including antioxidant, anti-inflammatory, and anticancer properties. However, the precise biochemical mechanisms responsible for its anticancer effects remain unclear, and the comprehensive composition of goji berry extracts is often insufficiently explored. This study aimed to investigate the biochemical pathways modulated in breast cancer cells by an ethanolic extract of *Lycium barbarum* fruit (LBE). Following metabolomic profiling using UHPLC-HRMS/MS, we assessed the antitumoral properties of LBE on different breast cancer cell lines. This investigation revealed that LBE exhibited cytotoxic effects, inducing a pro-oxidant effect that triggered pyroptosis activation through endoplasmic reticulum (ER) stress and subsequent activation of the P-IRE1α/XBP1/NLRP3 axis in MCF-7 cells. In addition, LBE did not display cytotoxicity toward healthy human cells but demonstrated antioxidant properties by neutralizing ROS generated by doxorubicin. These findings underscore the potential of LBE as a highly promising natural extract in cancer therapy.

## 1. Introduction

Cancer is the second leading cause of death worldwide and a major barrier to increasing life expectancy in every country in the world [1]. In fact, in 2020 alone, it caused approximately 5.5 million deaths among men and 4.4 million among women. Specifically, it is estimated that in 2022, breast cancer was the second most diagnosed cancer globally, accounting for 11.6% of all cases worldwide. It is the fifth leading cause of cancer death worldwide, with a mortality rate of 685,000 deaths annually and causing 6.9% of total cancer deaths [2,3].

Different therapeutic strategies have been explored, including surgery, radiotherapy, as well as chemotherapy, which are mainly part of standard protocols to produce adequate responses in most cases, but they can also exert significant adverse effects. Indeed, a multitude of chemotherapeutic agents manifest adverse side effects, with some presenting the risk of potentially irreversible chronic toxicity [4]. Therefore, there is an urgent need to explore novel therapeutic strategies and potential chemotherapy candidates. Nowadays, great attention has been paid to plant-derived antioxidant compounds with potential onconutraceutical properties [5]. In this regard, carotenoids and polyphenols, including phenolic acids and flavonoids, represent a large family of phytochemicals ubiquitous in nature and with significant antioxidant activity [6,7,8]. Their association with the health benefits derived from consuming substantial quantities of fruits and vegetables has been established. Regular consumption of dietary polyphenols and carotenoids has been linked to a reduced risk of chronic disorders related to oxidative stress, including neurodegenerative, cardiovascular, and inflammatory diseases. These beneficial effects stem from their ability to scavenge reactive oxygen species (ROS) [9,10,11], chelate toxic transitional metals inducing ROS, modulate the response of antioxidant enzymes [10,11], regulate redox balance, and maintain homeostasis in cells [2]. Furthermore, it has been widely demonstrated that dietary polyphenols and carotenoids are able not only to reduce oxidative and inflammatory stress, but also to decrease the proliferation of cancer cells [12], playing an essential role in chemoprevention. Several epidemiological studies have shown that the incidence of certain cancers such as breast cancer, colon cancer, prostate cancer, and lung cancer is lower in people who have a high consumption of fruits, vegetables, and beverages [13]. This suggests that plant-based diets are important in the prevention of breast cancer thanks to the presence of both nutritional bioactive compounds and non-nutritional ones like phenolic compounds. However, the results for individual supplements are still insufficient to fully validate their role in the prevention or treatment of breast cancer. This may be because the exact mechanisms of the bioactive compounds described in preclinical studies remain frequently unclear [14,15,16]. Therefore, this study, in line with other studies on the chemopreventive and/or chemosensitizing effects of dietary bioactive compounds on breast cancer, may help clarify the effects attributed to plant-based foods [16].

Specifically, various studies have focused their attention on the dual behavior of dietary polyphenols and carotenoids. These compounds are frequently utilized to shield the body from oxidative stress, but under specific conditions, they may display pro-oxidant properties. Factors such as the pH, phenolic concentrations, number, and position of hydroxyl groups in the aromatic rings, and even the presence of redox-active transition metals like Fe^3+^ and Cu^2+^ in cancer cells, are known to influence the conversion of phenolic compounds into oxidizing agents [17,18,19,20]. On the other hand, carotenoids have high reactivity due to their system of conjugated double bonds, being readily oxidized into oxidation products. When high amounts of oxygen are present, the carotenoid radical may react with O_2,_ generating a peroxyl radical which may promote lipid oxidation and oxidative damage in other biomolecules [21]. Given that the tumoral microenvironment is characterized by increased oxidative stress due to various factors including inflammation, alterations in metabolism, and hypoxia [22,23], antioxidants may assume a crucial role exploiting their selective pro-oxidant action in cancers as Trojan horses. This distinctive characteristic allows them to elevate oxidative stress to a level that limits tumor development [24,25,26,27]. Moreover, they play a role in mitigating oxidative damage in healthy cells, protecting these cells, and potentially preventing the initiation of tumorigenesis [28,29].

The direct pro-oxidant actions of phenolic compounds are based on the generation of a phenoxy radical or a redox complex with a transition metal ion. In detail, phenolic compounds undergo oxidation to form phenoxy radicals while simultaneously reducing transition metal ions. These phenoxy radicals can react with oxygen, leading to the generation of superoxide anions (O_2_·^−^). The produced superoxide anions then interact with phenolic compounds, producing hydrogen peroxide (H_2_O_2_). In the presence of reduced metal ions, hydrogen peroxide can further generate hydroxyl radicals (OH·) through a Fenton-like reaction. Hydroxyl radicals are notably the most potent oxidizing agents among all ROS produced in cells. Additionally, hydrogen peroxide may be produced by the dismutation of superoxide [30]. It is hypothesized that the pro-oxidant activity of several phenolic compounds can induce lipid peroxidation, DNA damage, and apoptosis in cancer cells [31].

ROS production has also been shown to initiate endoplasmic reticulum (ER) stress [32,33]. The ER is an essential cellular organelle network consisting of a series of tubules through which protein folding, lipid synthesis, the transport of cargo protein, and calcium storage are finely controlled [32]. When ER functions are impaired due to different physio-pathological phenomena, such as an increase in ROS, ER stress occurs [34]. The redox state is in fact closely linked to protein-folding homeostasis within the ER. Disulfide bond formation in the ER lumen is strongly dependent on redox balance, where this imbalance can disrupt protein folding and cause ER stress. Consequently, ROS-mediated ER stress triggers a cellular stress response, also defined as the unfolded protein response (UPR). The activation of the UPR involves three signaling pathways mediated by three transductors, PERK, IRE1, and ATF6, which all play vital functions in the overall recovery of protein misfolding. However, when the ER stress entity is too strong, the UPR directs the cell toward cell death [35,36,37,38]. In this context, numerous natural compounds have been identified for their ability to induce cell death in cancer cells by triggering ER stress through the excessive production of ROS.

Considering that cancer cells are characterized by higher levels of transition metal ions due, at least in part, to the overexpression of transferrin receptor and copper transporter 1, the use of plant-derived compounds with dual anti- and pro-oxidant properties represents an attractive strategy against cancer. This approach, commonly known as “oxidation therapy”, provides the advantage of minimal side effects due to the selective cytotoxicity of phytochemicals that specifically target cancer cells [39].

For these reasons, in the present study, we addressed how the dual action of certain components found within the fruits of *Lycium barbarum* can be considered to fight breast cancer cells. In fact, a recent study explored the dual nature of *Lycium barbarum* (Goji berry) which exhibited pro-oxidant properties in nematodes [40]. The fruits of *L. barbarum* L., known as goji berries or wolfberries, have been widely used as a traditional medicinal plant or dietary supplement in China for many years. It has also been found in the Mediterranean area with defoliated shrubs 1–2 cm long and bright red–orange ellipsoid berries [41]. *L. barbarum* fruit constituents include polysaccharides and proteoglycans, carotenoids, vitamins, fatty acids, free amino acids, flavonoids, phenolic acids, and anthocyanins [42,43]. There are numerous potential biological effects of the consumption of *Lycium* spp. [44], such as anti-inflammatory, anticancer, anti-obesity, antidiabetic, and cardiovascular protective effects [45,46,47,48].

Several studies have reported that the anticancer properties of *L. barbarum* are associated with its polysaccharide content [49,50]. In this study, we investigated, for the first time, the biochemical mechanisms underlying the anticancer properties of a phytocomplex extracted from *L. barbarum* fruit (LBE). Following analytical characterization, we assessed the anticancer properties of LBE on the breast cancer cell line MCF-7. Notably, we explored its pro-oxidant action at the molecular level by activating ER stress, suggesting a potential mechanism of tumor pyroptosis activation. Concurrently, we demonstrated its antioxidant efficacy on the MCF-10A healthy compartment, positioning it as a potent bifunctional onconutraceutical.

## 2. Materials and Methods

### 2.1. Sample Preparation

Red goji berries (*L. barbarum* L.) were kindly donated by the company DO.DA.CO. Srl (Scafati, Salerno, Italy). The fruits were freeze-dried for 24 h by setting the condenser temperature to −52 °C and the vacuum value to 0.100 mBar (LyoQuest-55, Telstar Technologies, Terrassa, Spain). The powder (1.0 g) was extracted for 20 min with 25 mL of 100% EtOH three times (last time overnight) at 40 °C under magnetic stirring at 470 rpm. The extract was centrifuged at 6000 rpm for 15 min at 4 °C, the supernatant was collected and filtered under a vacuum, and subsequently, the organic solvent was removed through vacuum evaporation using an Eppendorf™ Concentrator plus/Vacufuge^®^ plus at 35 °C and in V-HV (vacuum–high vapor) mode. Finally, in a separating funnel, the extract was dissolved in 50 mL of 100% 1-butanol and washed three times with water to remove the sugar. The organic phase was then collected, filtered on a 0.45 µm nylon membrane (Merck Millipore, Darmstadt, Germany), removing the solvent using a rotary evaporator, and then freeze-dried for 24 h

### 2.2. Determination of Total Phenolic Content (TPC), Total Flavonoid Content (TFC), and Total Chlorophyll and Carotenoid Content

The TPC of LBE was determined using the Folin–Ciocalteu method as described by Aquino G. et al. [51]. Gallic acid was chosen as standard for the quantification of TPC. A stock solution was prepared in 100% MeOH, and a calibration curve was generated within a concentration range of 10–500 μg/mL, with six concentration levels (y = 1779.61x − 2.0811; R^2^ = 99.92%). The TPC was expressed as milligrams of gallic acid equivalents per gram of dry weight (mg GAE/g DW).

The TFC was determined by applying the method reported by Imeneo, V. et al. [52]. The results were expressed as milligrams of rutin equivalents per gram of dry weight (mg RE/g DW) (10–500 μg/mL; y = 2239.24x − 5.3342; R^2^ = 99.93%).

The chlorophyll content was determined by using the methods previously described by Wang, H. et al. and Zhang, M. et al., and the carotenoid content was determined by using the protocol reported by Samec, D. et al. [53,54,55]. The content of the pigments was calculated based on the measured absorbance value, the solution volume, and the sample mass according to the following equations:

$$\mathrm{Chl}\;a\;(\mathrm{mg}/\mathrm{gDW})=\left\{\left[\left(12.25\times A\_663.2\right)-\left(2.79\times A\_646.8\right)\right]\times V\right\}/m$$

$$\mathrm{Chl}\;b\;(\mathrm{mg}/\mathrm{gDW})=\left\{\left[\left(21.50\times A\_646.8\right)-\left(5.10\times A\_663.2\right)\right]\times V\right\}/m$$

$$\mathrm{Car}\;(\mathrm{mg}/\mathrm{gDW})=\left\{\left[\left(4.75\times A\_452.5\right)-\left(0.226\times \left(Chl\;a+Chl\;b\right)\right)\right]\times V\right\}/m$$

where *V* is the volume of the extract (mL); *m* is the weight of the dry sample (mg); and A_663.2, A_646.8, and A_452.5 are the absorbance values of the mixture solution at 663.2, 646.8, and 452.5 nm, respectively.

### 2.3. Determination of Antioxidant Activity through DPPH and ABTS Assays

The antioxidant activity of LBE was assessed through two radical scavenging tests: the 1,1-diphenyl-2-picrylhydrazyl (DPPH) assay [56] and 2,2′-azino-bis-3-ethylbenzthiazoline-6-sulphonic acid (ABTS) assay [57]. The results from both assays were expressed as an IC_50_ value (µg/mL); a lower IC_50_ value represents a stronger DPPH and ABTS scavenging capacity. Trolox was used as the positive antioxidant control and the results were also expressed as an IC_50_ value (µg/mL).

### 2.4. Metal Binding Studies

The metal binding studies were performed as described in [58]. The UV absorption of the LBE (500 μg/mL) alone or in the presence of CuSO_4_, FeSO_4,_ or FeCl_3_ (40 μM) for 30 min in 20% (*v*/*v*) ethanol/buffer (20 mM HEPES, 150 mM NaCl, pH 7.4) was recorded using a microplate reader (Multiskan Go, Thermo Scientific, Waltham, MA, USA) with a wavelength ranging from 280 to 400 nm. The final volume of the reaction mixture was 1 mL.

### 2.5. UHPLC-HRMS/MS Analysis

LC-MS/MS analysis was performed on a Thermo Ultimate RS 3000 coupled online to a Q-Exactive hybrid quadrupole Orbitrap mass spectrometer (Thermo Fisher Scientific) equipped with a heated electrospray ionization probe (HESI II).

Separation was performed in reversed-phase mode, with a Kinetex^®^ 2.6 µm EVO C18 100 Å and an LC Column 150 × 2.1 mm (Phenomenex, Bologna, Italy) with a guard cartridge system (SecurityGuard ULTRA cartridges for EVO-C18, sub-2 µm and core–shell columns with 2.1 mm internal diameters). The column temperature was set at 40 °C and the flow rate was 0.4 mL/min. The mobile phase was (A) H_2_O with 0.1% HCOOH (*v*/*v*) and (B) ACN with 0.1% HCOOH (*v*/*v*). The following gradient was employed: 0.01–3.00 min, isocratic to 0% B; 3.01–10.00 min, 0–10% B; 10.01–25.00 min, 10–20% B; 25.01–30.00 min, 20–50% B; 30.01–33.00 min, 50–95% B; 33.01–36.00 min, isocratic to 95% B; 36.01–38.00 min, 95–0%; then, six minutes were employed for column re-equilibration. The injection volume was 5 µL. All additives and mobile phases were LCMS grade and purchased from Merck (Milan, Italy).

The ESI was operated in positive and negative mode. The MS was calibrated by Thermo CalMix Pierce™ calibration solutions in both polarities. Full MS (100–1500 *m/z*) and data-dependent MS/MS were performed at a resolution of 35,000 and 17,500 FWHM, respectively, and normalized collision energy (NCE) values of 15, 20, and 25 were used. The source parameters were as follows: sheath gas pressure, 50 arbitrary units; auxiliary gas flow, 13 arbitrary units; spray voltage, +3.5 kV, −2.8 kV; capillary temperature, 310 °C; auxiliary gas heater temperature, 300 °C.

Identification of the investigated analytes was carried out by comparing their retention times and MS/MS data with those present in the literature.

The MS spectra were processed using FreeStyle™ 1.8 SP2 and the commercial software Compound Discoverer v. 3.3.1.111 SP1 (Thermo Fisher, Bremen, Germany). Identification was accomplished by activating the Chem Spider and mzCloud nodes. The following online databases were also consulted accessed, on 1 September 2023: Phenol-Explorer (www.phenolexplorer.eu), PubChem (https://pubchem.ncbi.nlm.nih.gov), and SciFinder Scholar (https://scifinder.cas.org).

### 2.6. Quantitative Analysis

The quantification of phytochemicals in the LBE extract was performed using LC-MS/MS.

For the quantitative analysis of hydroxycinnamic acids, flavonoids, and fatty acids, we used as an external standard caffeic acid (CA), rutin (R), and oleic acid (OA) in negative ionization mode, respectively.

Stock solutions were prepared in MeOH, and the calibration curves were obtained in a concentration range of 0.03–3.91 µg mL^−1^ for caffeic acid, 0.06–15.63 µg mL^−1^ for rutin, and 15.63–2000 µg mL^−1^ for oleic acid, using six concentration levels with triplicate injections for each level. Linear regression was used to generate the calibration curves with R^2^ values ≥ 0.999. Extracted ion chromatogram (XIC) areas of the standard were plotted against corresponding concentrations (µg/mL^−1^). The compound content in the sample was expressed as milligrams of oleic acid (OAE), micrograms of rutin (RE), and micrograms of caffeic acid equivalent (CAE) per gram of dried extract.

The method validation parameters for the quantitative assay included accuracy, linearity, range, limit of detection (LOD), and limit of quantitation (LOQ). LOD and LOQ were calculated by using the standard deviation (SD) and the slope of the calibration curve, multiplied by 3.3 and 10, respectively (Table S1).

### 2.7. Cell Cultures and Drug Treatment

The human breast cancer cell lines, MCF-7, SK-BR-3, and MDA-MB-231, were obtained from American Type Culture Collection (ATCC, Rockville, MD, USA). Cells were grown in Dulbecco’s Modified Eagle Medium (DMEM, 4500 mg/mL glucose) supplemented with 10% (*v*/*v*) fetal bovine serum, 2 mM L-glutamine, 100 U/mL penicillin, and 0.1 mg/mL streptomycin.

The human breast endothelial line (MCF-10A) was purchased from ATCC and maintained in a 1:1 mixture of DMEM and Ham’s F12 medium supplemented with 5% horse serum, 2 mM L-glutamine, human recombinant epidermal growth factor (20 ng/mL), insulin (10 mg/mL), cholera toxin (100 ng/mL), and hydrocortisone (5 mg/mL).

Cells were routinely grown in culture dishes (Corning, Corning, NY, USA) in a 95% humidified environment containing 5% CO_2_ at 37 °C and split every 2 days. In each experiment, cells were placed in a fresh medium and cultured in the presence of LBE at different concentrations and times, as reported in subsequent sections. Each treatment and analysis were performed at least in three separate experiments.

### 2.8. Cell Viability Assay and Phase-Contrast Analysis

Cell viability was established by measuring the mitochondrial metabolic activity with the MTT assay [59,60]. Briefly, MCF-7 (6 × 10^3^ cells/well), MDA-MB-231 (6 × 10^3^ cells/well), SK-BR-3 (6 × 10^3^ cells/well), and MCF-10A (6 × 10^3^ cells/well) were plated into 96-well plates, and then, LBE (3.125–100 µg/mL) was added for 24 h. Afterward, the MTT reagent was added for 2–4 h depending on the cell line. Cell lysis was performed with an isopropanol/HCl solution to dissolve blue formazan crystals formed by viable mitochondria. The absorbance of formazan crystals was measured at 570 nm (Multiskan Go, Thermo Scientific, Waltham, MA, USA). Phase-contrast images were captured using a Zeiss Axiocam 208 inverted microscope (40× objective) (Carl Zeiss Microscopy Ltd., Jena, Germany).

### 2.9. Colony Formation Assay

The clonogenic potential was assessed using 6 µg/mL of LBE, as performed previously [9]. Cells were plated in 6-well plates at a seeding density of 5 × 10^2^ cells/well. After incubation for 7 days, cells were fixed and stained with a solution containing 3.7% formaldehyde and 0.5% crystal violet for 30 min. Images were obtained and the clonogenic potential was determined from a 1% SDS cell dissolution and measured by using a spectrophotometer at 570 nm.

### 2.10. Wound Healing Assay

In the wound healing analysis, 3 × 10^5^ cells were seeded in 6-well plates and then incubated at 37 °C for 24 h. After that, a linear scratch was created with a 10 µL sterile pipette tip. Cells were washed with PBS and cultured in a medium containing 6 µg/mL of LBE. To avoid cell proliferation, DMEM with 2% FBS was used. Different fields were analyzed by contrast-phase microscopy, and each scratch area was photographed at 0 and 24 h. Images were obtained, and the wound size area (%) was calculated using ImageJ, version 1.47.

### 2.11. Determination of Hypodiploid Nuclei

Hypodiploid nuclei were analyzed by using propidium iodide (PI) staining and flow cytometry, as described previously [61]. MCF-7 cells (4 × 10^4^ cells/well) were grown in 12-well plates and allowed to adhere for 24 h. Next, the cells were treated with LBE (25, 12, 6 µg/mL) for 24 h. After treatments, the culture medium was replaced, and the cells were washed and suspended in PI buffer. After incubation at 4 °C for 30 min in the dark, cell nuclei were analyzed with a Becton Dickinson FACScan flow cytometer using the Cell Quest software, version 4 (Franklin Lakes, NJ, USA).

### 2.12. PI/Hoechst 33342 Double Staining Assay

Cell-permeable DNA dye Hoechst 33342 and PI were used to validate necrosis in cell populations. MCF-7 cells (4 × 10^4^ cells/well) seeded on glass cover slips were grown in 12-well plates and allowed to adhere for 24 h. Next, the medium was replaced, and the cells were treated with LBE (25, 12, 6 µg/mL) for 24 h. After treatments, the culture medium was replaced, and live cells were stained with a Hoechst 33342/PI solution added to the cell culture medium (Hoechst 33342, 5 µM; PI, 30 µg/mL) at 37 °C for 20 min in the dark [62]. The stained cells were washed two times and then fixed with 3.7% formaldehyde for 10 min. Images were acquired on a fluorescence microscope (Axioshop 40, Zeiss; magnification, 20×). Quantitative analyses were performed by using the ImageJ program, version 1.47 (N ≥ 10), and expressed as PI-positive cells (%).

### 2.13. Western Blotting Analysis

The MCF-7 cell line was seeded in 60 mm culture dishes and treated with LBE (6 μg/mL) at different times. After 8 h, the cells were washed, detached with a scraper, and centrifuged to remove debris. Full proteins were extracted by using a lysis buffer. Then, cell lysates were centrifuged at 4850× *g* for 20 min at 4 °C. A total of 30 μg of total proteins was run on 8–12% SDS-PAGE and transferred to nitrocellulose membranes using a minigel apparatus (Bio-Rad Laboratories, Hercules, CA, USA). Blots were blocked in phosphate-buffered saline, containing Tween-20 0.1% and 10% non-fat dry milk, for 1 h at room temperature and incubated overnight with specific primary antibodies at 4 °C with slight agitation. α-tubulin was used as the loading control. The following antibodies were used: rabbit monoclonal anti-PERK (Cell Signaling, Danvers, MA, USA), mouse monoclonal anti-ATF6 (Cell Signaling), rabbit polyclonal anti-caspase-12 (Abcam, Cambridge, UK), mouse monoclonal anti-α-tubulin (Santa Cruz Biotechnology, Dallas, TX, USA), rabbit polyclonal anti-phospho-IRE1α (Sigma Aldrich), mouse monoclonal anti-NRLP3 (Sigma Aldrich), rabbit polyclonal anti-GRP78 (Sigma Aldrich), rabbit monoclonal anti-Nrf2 (Santa Cruz Biotechnology), rabbit polyclonal anti-CHOP (Sigma Aldrich), rabbit polyclonal anti-caspase-3 (Cell Signaling), and mouse monoclonal anti-caspase-1 (Santa Cruz Biotechnology). After washing, peroxidase-linked secondary antibody (Pierce, Thermo Fisher Scientific) was added for 1 h at room temperature [63]. Antigen–antibody complexes were detected through enhanced chemiluminescence using LAS 4000 (GE Healthcare, Chicago, IL, USA) and a densitometry analysis of the autoradiographs was performed by using the ImageJ program, version 1.47.

### 2.14. Measurement of LDH

To verify the release of LDH into the cell culture medium after plasma membrane disruption, the LDH-Glo™ cytotoxicity assay (Promega, Madison, WI, USA) was performed. According to the LDH-Glo™ kit protocol, the LDH detection reagent (containing lactate, NAD^+^, reductase, reductase substrate, and rLuciferase Ultra-Glo™) were added to the cell culture medium sample. The luminescent signal generated was read in end-point mode using a PerkinElmer AlphaScreen multimode plate reader.

### 2.15. ROS Detection

Reactive oxygen species (ROS) levels were measured by using 10 μM 6-carboxy-2′,7′-dichlorodihydrofluorescein diacetate (DCFH-DA, Sigma Aldrich, St. Louis, MO, USA). MCF-7 cells were seeded (6 × 10^3^ cells/well) in a black 96-well ViewPlate (PerkinElmer, Waltham, MA, USA), allowing them to adhere for 24 h. Next, the cells were incubated for 1–24 h with 6 µg/mL LBE. Doxorubicin (400 nM, 4 h) was used as the positive control. After washing, a staining solution containing DCFH-DA in serum-free medium without phenol red was added for 40 min at 37 °C in the dark. The fluorescence signals (excitation/emission 485 nm/535 nm) were read using a PerkinElmer EnSpire multimode plate reader and expressed as DCFH fluorescence intensity.

### 2.16. RNA Extraction, Reverse Transcription, and Real-Time PCR

Total RNA was isolated from the treated cells after 24 h using Trizol reagent (Gibco, Thermo Fisher Scientific), according to the manufacturer’s instructions. Aliquots of total RNA for the real-time PCR test were subjected to DNase I digestion (Thermo Fisher Scientific) and were reverse-transcribed using M-MLV Reverse Transcriptase (Thermo Fisher Scientific) according to the manufacturer’s protocol. The thermal conditions for reverse transcription were 25 °C for 10 min, 37 °C for 50 min, and 75 °C for 15 min. In the last step, RNAse H was added.

Real-time PCR was performed with the QuantStudio™ 5 Real-Time PCR System (Thermo Fisher Scientific) using SYBR Green detection in a total volume of 20 μL with 1 μL of forward and reverse primers (5 μM) and 10 μL of PowerUp™ SYBR™ Green Master Mix (Thermo Fisher Scientific). Reactions included an initial cycle at 50 °C for 2 min and 95 °C for 2 min, followed by 40 cycles of denaturation at 95 °C for 15 s, annealing at 60 °C for 15 s, and extension at 72 °C for 1 min. The values were determined from a standard curve generated from serial cDNA dilutions and normalized to GAPDH.

The primers used for the qPCR reactions were as follows: forward ATF4 5′-GTC CCT CCA ACA ACA GCA AG-3′; reverse ATF4 5′-CTA TAC CCA ACA GGG CAT CC-3′; forward Xbp-1 5′-TTA CGA GAG AAA ACT CAT GGC-3′; reverse Xbp-1 5′-GGG TCC AAG TTG TCC AGA ATG C-3′; forward SOD1 5′-AAGGCCGTGTGCGTGCTGAA-3′; reverse SOD1 5′-CAGGTCTCCAACATGCCTCT-3′; forward Catalase 5′-GCAGATACCTGTGAACTGTC-3′; reverse Catalase 5′-GTAGAATGTCCGCACCTGAG-3′. The 2^−ΔΔCT^ method was used to analyze the results and relative mRNA expression levels were determined as the fold-induction relative to Ctrl cells, set as 1.

### 2.17. RT-PCR and XBP1 Splicing Assay

MCF-7 cells were seeded in 100 mm culture dishes and treated alone with LBE (6 μg/mL). Thapsigargin was used as the positive control for XBP1 splicing. After 24 h, the total RNA of MCF-7 cells was extracted. The protocol conducted was previously reported by Vestuto et al. [9]. Ethidium bromide-stained amplicons were exposed to LAS 4000 (GE Healthcare).

### 2.18. Determination of Protein Misfolding

Protein misfolding was analyzed by using Thioflavn T (ThT, Sigma Aldrich, St. Louis, MO, USA) staining and flow cytometry. MCF-7 cells (20 × 10^3^ cells/well) were grown in 24-well plates and allowed to adhere for 24 h. Later, the medium was replaced, and the cells were treated with LBE (25 µg/mL) and also thapsigargin (300 nM) as the positive control for 24 h. After treatments, the culture medium was replaced, and the cells were washed and suspended in 100 μL ThT (final concentration, 20 µM). After incubation at 4 °C for 30 min in the dark, the cells were analyzed with the same flow cytometer reported above. A second analysis was performed through fluorescence microscopy. MCF-7 cells (4 × 10^4^ cells/well) seeded on glass cover slips were grown in 12-well plates and allowed to adhere for 24 h. After treatments, live cells were stained with a Hoechst 33342/ThT solution added to the cell culture medium (Hoechst 33342, 5 µM; ThT, 20 µM) at 37 °C for 20 min in the dark. The stained cells were washed and fixed with 3.7% formaldehyde for 10 min. Images were acquired using a fluorescence microscope (Magnification, 20×). Quantitative analyses were performed by using the ImageJ program, version 1.47 (N ≥ 10), and expressed as ThT-positive cells (%).

### 2.19. Measurement of Intracellular Ca^2+^ Signaling

Intracellular Ca^2+^ concentrations were measured according to Di Sarno et al., with minor modifications [64]. The fluorescent Mag Fluo-4 a.m. probe (Molecular Probes, Thermo Fisher Scientific) was used. MCF-7 cells (20 × 10^4^ cells/well) were grown in 6-well plates and allowed to adhere for 24 h. Later, the medium was replaced, and the cells were treated with LBE (25 µg/mL) for 24 h. After treatments, the cells were trypsinized and incubated with Mag-Fluo-4 a.m. (22 μM final concentration) in DMEM without serum for 1 h at RT. Then, the cells were washed through centrifugation and incubated at 37 °C for 20 min in PBS (without calcium and magnesium). Finally, the cells were washed and the fluorescence in each sample was analyzed by flow cytometry.

### 2.20. Statistical Analysis

The data are reported as mean ± SD of the results from three independent experiments. Statistical analysis was performed using an analysis of variance test (ANOVA), and multiple comparisons were made with the Bonferroni test using GraphPad Prism 8.0 software (San Diego, CA, USA). Significance was assumed at *p* < 0.05.

## 3. Results

### 3.1. Total Phenolic, Flavonoid, Chlorophyll, and Carotenoid Content, and Antioxidant Activity

This study aimed to assess the phenolic, flavonoid, chlorophyll, and carotenoid composition of LBE. Our findings indicate that LBE possesses TPC and TFC values of approximately 31.26 ± 0.01 mg GAE/g DW and 65.51 ± 0.01 mg RE/g DW, respectively. The values investigated in this study were comparable to those stated by Wang et al. [65] for TFC and TPC in different goji genotypes.

Furthermore, spectrophotometric analysis revealed the presence of chlorophyll a and chlorophyll b at concentrations of 0.417 ± 0.001 mg/g DW and 0.693 ± 0.019 mg/g DW, respectively. Additionally, in agreement with the results reported by Ilić et al., the total carotenoid content in the ethanolic extract was quantified as 0.508 ± 0.004 mg/g DW [66].

The antioxidant potential of LBE was evaluated through the DPPH and ABTS assays. In the ABTS assay, a blue/green ABTS^·+^ radical is formed and can be mitigated by antioxidants, while the DPPH assay involves the reduction of purple DPPH to 1,1-diphenyl-2-picryl hydrazine.

The DPPH free radical scavenging activity of LBE yielded an IC_50_ value of 533.17 ± 3.32 µg/mL (Trolox, IC_50_ of 4.57 ± 0.27 µg/mL), which, when compared with values reported by Skenderidis et al., indicated a notable antioxidant activity [67].

Furthermore, LBE exhibited good ABTS scavenging potency (IC_50_ 9.05 ± 1.73 µg/mL), showing 20 times less activity than the positive control (Trolox, IC_50_ of 0.45 ± 0.04 µg/mL), consistent with previously reported results [68].

Metal binding studies were performed to further support these results. The results showed that LBE also exerted its antioxidant activity through chelation with iron and copper ions (Figure S1), in agreement with the high polyphenolic content and DPPH and ABTS scavenging action determined through previous assays.

### 3.2. Phytochemical Profile of LBE

Assessment of the metabolomic profile of LBE was carried out through LC-MS/MS analysis. Operating in negative and positive ionization mode, the analytical platform allowed us to tentatively identify 60 different compounds (Table 1). Notably, LC-MS/MS analysis revealed the presence of diverse classes of free polyphenols, including phenolic acids, hydroxycinnamic acids, and flavonoids.

Concerning hydroxycinnamic acids, different compounds containing various groups were detected, including caffeoyl (C, 162 Da), feruloyl (f, 175 Da), coumaroyl (c, 162 Da), spermidine (s, 145 Da), putrescine (p, 90 Da), and tyramine (t, 137 Da).

According to the negative fragmentation pattern, peaks **14** and **15** were tentatively identified as *N^1^*-dihydrocaffeoyl, *N^10^*-caffeoyl spermidine, with a molecular ion at *m*/*z* 470 corresponding to [C_25_H_33_N_3_O_6_]^−^. The MS^2^ spectra showed the presence of *m*/*z* 308 and *m*/*z* 163 product ions, corresponding to the loss of a caffeoyl group (162 Da, C_9_H_8_O_3_) and a subsequent loss of a spermidine amide moiety (145 Da, C_7_H_19_N_3_) [69].

Peak **11** exhibits [M+H]^+^ ions at *m*/*z* 634 (C_31_H_44_O_11_N_3_) and fragment ions at *m*/*z* 472 [M–H–Hex]^+^, *m*/*z* 310 [M–H–Hex–C]^+^, *m*/*z* 220 [M–H–Hex–C–p]^+^, and *m*/*z* 163 [M–H–Hex–C–s]^+^. This suggests a tentative identification as *N^1^*-dihydrocaffeoyl, *N^10^*-caffeoyl spermidine hexose (Figure S2) [70]. Peak **6** was tentatively identified as di-*O*-caffeoylquinic acid, with an [M–H]^−^ ion observed at *m*/*z* 515. Its MS/MS fragmentation pattern was characterized by a base peak at *m*/*z* 353 and *m*/*z* 323, corresponding to the loss of caffeoyl [M–H–162]^−^ and quinic moieties [M–H–192]^−^.

Peak **9** exhibited an [M–H]^−^ ion at *m/z* 515, along with main fragment ions at *m*/*z* 191 [M–H–162–162]^−^ and *m*/*z* 179 [M–H–162–18]^−^. These ions correspond to the loss of a dicaffeoyl moiety and water molecules, respectively. Consequently, peak **9** was identified as di-*O*-caffeoylquinic acid derivatives (7.68 ± 1.98 µg CAE g^−1^ dw) [71].

Peaks **29** and **37** (53.05 ± 3.07 µg CAE g^−1^ dw) exhibited [M–H]^−^ ions at *m*/*z* 282 (C_17_H_17_NO_3_) with a fragment ion at *m/z* 119 [M–H–c]^−^. These peaks were identified as isomers of N-*p*-coumaroyl tyramine. The same compound (**38**) was also detected in positive ionization mode, producing [M–H–t]^−^ and [M–H–c]^−^ ions, confirming the presence of both coumaroyl and tyramine units (Figure S3) [72].

Further hydroxycinnamic acids were found and peaks **7** (1.068 ± 0.12 µg CAE g^−1^ dw), **16** (214.43 ± 1.42 µg CAE g^−1^ dw), and **23** (0.998 ± 0.18 µg CAE g^−1^ dw) were tentatively identified as caffeic acid (*m*/*z* 179, C_9_H_8_O_4_), *p*-coumaric acid (*m*/*z* 163, C_9_H_8_O_3_), and ferulic acid (*m*/*z* 193, C_10_H_10_O_4_), respectively. Their fragmentation pattern was mainly characterized by CO_2_ neutral loss (44 Da), resulting in MS^2^ ions at *m*/*z* 135 (peak **7**), *m*/*z* 119 (peak **16**), and *m*/*z* 149 (peak **23**). Additionally, the loss of a CH_3_ residue (15 Da) followed by the removal of CO_2_ (44 Da) generated ferulic acid fragments at *m*/*z* 178 [M–H–CH_3_]^−^ and *m*/*z* 134 [M–H–CO_2_]^−^.

Numerous flavonoids have been identified in LBE, primarily corresponding to rutin (*m/z* 609), isorhamnetin (*m*/*z* 316), quercetin (*m*/*z* 303), and kaempferol (*m*/*z* 287) aglycones.

Compound **21** (18.49 ± 7.96 µg RE g^−1^ dw) was tentatively identified as rutin hexoside (quercetin-*O*-Rham-Hex-*O*-Hex) with an ion at *m*/*z* 771 [M–H]^−^. Its MS/MS fragmentation pattern was characterized by *m/z* 609 [M–H–162]^−^, *m/z* 462 [M–H–162–147]^−^, and *m*/*z* 301 [M–H–162–146–162]^−^, indicating successive losses of hexosyl, rhamnosyl, and hexosyl groups.

Rutin aglycone (*m/z* 609) was also identified as compound **26** (1607 ± 89.85 µg RE g^−1^ dw) and **27 [69]**. Similarly, peaks **31** (298.88 ± 17.93 µg RE g^−1^ dw) and **32** were tentatively identified as kaempferol *O*-hexoside-rhamnoside, also known as nictoflorin (C_27_H_30_O_15_). They exhibited signals corresponding to [M–H]^−^ at *m/z* 593 and [M+H]^+^ at *m/z* 595. The key product ions in the negative and positive ionization modes at *m*/*z* 285/287 correspond to the loss of the rhamnosyl and glucosyl units (Figure S4) [63].

The MS spectra showed a deprotonated molecular ion at *m*/*z* 623, corresponding to the molecular formula C_28_H_32_O_16_. However, the loss of hexoside (162 Da, C_6_H_10_O_5_) and rhamnoside (146 Da, C_6_H_10_O_4_) moieties, followed by the sequential loss of methyl residue (15 Da, CH_3_), produced fragments at *m*/*z* 315 [M–H–C_6_H_10_O_5_–C_6_H_10_O_4_]^−^ and *m*/*z* 300 [M–H–C_6_H_10_O_5_–C_6_H_10_O_4_–CH_3_]^−^. This led to the tentative identification as isorhamnetin-*O*-rutinoside (peak **35**, 182.55 ± 36.32 µg RE g^−1^ dw).

Peak **36** was already identified as isorhamnetin-*O*-hexoside (*m*/*z* 477, C_22_H_22_O_12_) based on its MS/MS fragments at *m/z* 314 [M–H–C_6_H_10_O_5_]^−^, *m*/*z* 271 [M–H–C_6_H_10_O_5_–CH_3_–CO]^−^, and *m*/*z* 243 [M–H–C_6_H_10_O_5_–CH_3_–2CO]^−^. These correspond to the relative loss of the sugar moiety (162 Da), methyl residue (15 Da), and CO (28 Da) units [71].

In LBE, hydroxy fatty acids were identified. Peak **52** exhibited the precursor ion at *m*/*z* 295. However, the loss of a water molecule [M–H–18]^−^ and the cleavage of the C=C bond at C9–C10 resulted in fragments at *m*/*z* 277 and 171, suggesting its tentative identification as hydroxy octadecadienoic acid (HODE) and quantified at 1737.23 ± 23.17 mg OAE g^−1^ dw (Figure S5) [73].

Furthermore, peaks **47**, **48,** and **49** were observed at different retention times; all of them exhibited [M–H]^−^ ions at *m*/*z* 311 and were quantified, respectively, at 538.99 ± 6.96 mg g^−1^ dw, 435.75 ± 2.64 mg OAE g^−1^ dw, and 433.99 ± 7.6 mg OAE g^−1^ dw. Their MS/MS fragmentation pattern was characterized by a base peak at *m*/*z* 293, derived from the loss of a water molecule [M–H–18]^−^. Thus, these compounds were tentatively identified as dihydroxy octadecadienoic acid (DiHODE) [74].

Additionally, isomers of trihydroxy octadecadienoic acid (TriHODE) were identified (peaks **42**, **44**, and **45**) with [M–H]^−^ ions at *m*/*z* 327 and product ions at *m*/*z* 309 and *m*/*z* 291. These ions derive from the loss of water units [M–H–18–18]^−^ [75,76,77]. Quantitative analysis gave the amount of each compound as 3.22 ± 0.3 mg OAE g^−1^ dw, 276.61 ± 6.2 mg OAE g^−1^ dw, and 440.45 ± 10.03 mg OAE g^−1^ dw, respectively (Table 2).

**Table 1:** Metabolomic profiling of LBE.

Peak	Compound	Rt (min)	[M–H]^−^	[M+H]^+^	MS/MS	Molecular Formula	Error (ppm)	Classification	Reference
**1**	Gallic acid	2.37	169.0136	-	125.0233	C_7_H_5_O_5_	1.55	Phenolic acids	[72]
**2**	*p*-coumaric acid-hexoside	3.41	325.0934	-	119.0491 163.0392	C_15_H_18_O_8_	0.76	Hydroxycinnamic acid derivates	[72]
**3**	Hydroxybenzoic acid	5.15	137.0235	-	93.0333	C_7_H_6_O_3_	0.74	Phenolic acids	[72]
**4**	Benzoic acid	6.68	121.0285	-	/	C_7_H_6_O_2_	−0.33	Organic acids	[72]
**5**	Blechnic acid	7.37	371.0988	-	119.0491 163.0392	C_16_H_20_O_10_	2.69	Flavonoids	[78]
**6**	di-*O*-caffeoylquinic acid	8.60	515.1413	-	353.0885 323.0759	C_22_H_28_O_14_	1.77	Hydroxycinnamic acid derivates	[72]
**7**	Caffeic acid	9.06	179.0344	-	135.0441	C_9_H_8_O_4_	1.13	Hydroxycinnamic acids	[72]
**8**	*p*-coumaric acid-hexoside (*isomer I*)	9.35	325.0934	-	145.0285 163.0391	C_15_H_18_O_8_	4.42	Hydroxycinnamic acid derivates	[72]
**9**	di-*O*-caffeoylquinic acid	10.10	515.1415	-	191.0554 179.0333	C_22_H_28_O_14_	3.78	Hydroxycinnamic acid derivates	[72]
**10**	*N*-Caffeoyl, *N′*-dihydrocaffeoyl spermidine dihexose	10.46	-	796.3496	163.0388 220.0966 310.2125 472.2498	C_37_H_53_N_3_O_16_	−1.31	Hydroxycinnamic acid amides	[65,70]
**11**	*N^1^*-dihydrocaffeoyl, *N^10^*-caffeoyl spermidine hexose	11.25	-	634.2977	163.0389 220.0967 165.0546 472.2523 310.2133	C_31_H_43_N_3_O_11_	−0.54	Hydroxycinnamic acid amides	[65,70]
**12**	Ferulic acid hexoside	11.26	355.1041	-	175.0392 160.0157	C_16_H_20_O_9_	6.70	Hydroxycinnamic acid derivates	[74]
**13**	*p*-coumaric acid-hexoside (*isomer II*)	11.40	325.0933	-	145.0285 163.0390 119.0491	C_15_H_18_O_8_	2.26	Hydroxycinnamic acid derivates	[72]
**14**	*N^1^*-dihydrocaffeoyl, *N^10^*-caffeoyl spermidine	11.76	470.2301	-	135.0442 308.1985 163.0402 291.1843	C_25_H_33_N_3_O_6_	−4.32	Hydroxycinnamic acid amides	[65]
**15**	*N^1^*-dihydrocaffeoyl, *N^10^*-caffeoyl spermidine (*isomer I*)	11.77	-	472.2445	163.0389 220.0967 310.2130 293.1865 236.1276	C_25_H_33_N_3_O_6_	1.08	Hydroxycinnamic acid amides	[65,70]
**16**	*p*-coumaric acid	11.88	163.0392	-	119.0490	C_9_H_8_O_3_	0.94	Hydroxycinnamic acids	[72]
**17**	*p*-coumaric acid (*isomer I*)	11.97	-	165.0547	147.0439 119.0492	C_9_H_8_O_3_	−0.96	Hydroxycinnamic acids	[79]
**18**	*N^1^,N^10^*-bis-(caffeoyl) spermidine dihexose	12.07	-	794.3350	163.0389 220.0969 308.1956 632.3118 470.2307	C_40_H_49_N_4_O_13_	−0.28	Hydroxycinnamic acid amides	[65,70]
**19**	*p*-coumaroyl-quinic acid	12.76	337.0935	-	191.0553 93.0333	C_16_H_18_O_8_	3.09	Hydroxycinnamic acid derivates	[72]
**20**	*O*-*trans*-feruloyl-*O*-*β*-d-glucopyranosyl-*α*-d-glucopyranoside	12.80	517.1569	-	193.0500 175.0392	C_22_H_30_O_14_	2.32	Hydroxycinnamic acid derivates	[72]
**21**	Rutin hexose	13.35	771.2005	-	609.1435 462.0828 301.0348	C_33_H_40_O_21_	2.72	Flavonoids	[74]
**22**	Scopoletin	13.70	191.0343	-	176.0107 148.0156	C_10_H_8_O_4_	2.18	Coumarins	[56]
**23**	Ferulic acid	14.11	193.0501	-	134.0363 178.0264 149.0598	C_10_H_10_O_4_	2.42	Hydroxycinnamic acids	[72]
**24**	*N*-acetyl-DL-tryptophan	14.23	245.0931	-	203.0820 74.0235 116.0493	C_13_H_14_N_2_O_3_	3.93	Tryptophan derivates	[74]
**25**	Azelaic acid	17.44	187.0968	-	125.0961 97.0646	C_9_H_16_O_4_	2.02	Fatty acids	[74]
**26**	Rutin	18.05	609.1463	-	300.0275 301.0356	C_27_H_30_O_16_	0.41	Flavonoids	[72]
**27**	Rutin (*isomer I*)	18.07	-	611.1603	303.0497 465.1002	C_27_H_30_O_16_	−0.63	Flavonoids	[79]
**28**	Quercetin-*O*-hexoside	18.24	463.0886	-	300.0275 301.0358	C_21_H_20_O_12_	2.69	Flavonoids	[72]
**29**	*N*-*p*-*cis/trans*-coumaroyl-tyramine	18.81	282.1139	-	119.0491 243.0793	C_17_H_17_NO_3_	4.28	Hydroxycinnamic acid amides	[72]
**30**	Naringenin-*O*-hexoside (Prunin)	18.99	433.1145	-	271.0610	C_21_H_22_O_10_	2.38	Flavonoids	[72]
**31**	Kaempferol *O*-hexoside-rhamnoside (Nictoflorin)	19.90	593.1516	-	285.0405 255.0298	C_27_H_30_O_15_	2.04	Flavonoids	[72]
**32**	Kaempferol *O*-hexoside-rhamnoside (Nictoflorin) (*isomer I*)	19.96	-	595.1658	287.0550 258.2194	C_27_H_30_O_15_	−0.37	Flavonoids	[79]
**33**	*N-cis/trans*-feruloyltyramine	20.05	312.1245	-	148.0520 297.1012	C_18_H_19_NO_4_	3.75	Hydroxycinnamic acid amides	[72]
**34**	Kaempferol-*O*-hexoside	20.06	447.0939	-	284.0340 285.0403 255.0299	C_21_H_20_O_11_	−0.87	Flavonoids	[72]
**35**	Isorhamnetin-*O*-rutinoside	20.45	623.1626	-	315.0511 300.0198	C_28_H_32_O_16_	2.13	Flavonoids	[72]
**36**	Isorhamnetin-*O*-hexoside	20.68	477.1046	-	314.0432 243.0302 271.0262	C_22_H_22_O_12_	1.09	Flavonoids	[72]
**37**	*N*-*p*-*cis/trans*-coumaroyl-tyramine (*isomer I*)	21.60	282.1139	-	119.0491	C_17_H_17_NO_3_	4.28	Hydroxycinnamic acid amides	[72]
**38**	*N*-*p*-*cis/trans*-coumaroyl-tyramine (*isomer II*)	21.63	-	284.1280	147.0439 121.0649 164.0692	C_17_H_17_NO_3_	−0.88	Hydroxycinnamic acid amides	[72]
**39**	*N*-*cis/trans*-feruloyltyramine	22.62	312.1245	-	148.0520 297.1007	C_18_H_19_NO_4_	4.15	Hydroxycinnamic acid amides	[72]
**40**	Quercetin	23.36	301.2023	-	151.0028 178.9983	C_15_H_10_O_7_	4.13	Flavonoids	[72]
**41**	Naringenin	23.82	271.0615	-	119.0491 151.0028	C_15_H_12_O_5_	5.03	Flavonoids	[72]
**42**	Trihydroxy-octadecadienoic acid (TriHODE)	24.46	327.2179	-	171.1019 211.1336 291.2002	C_18_H_32_O_5_	4.33	Fatty acids	CD
**43**	Trihydroxy-octadecenoic acid	24.84	329.2334	-	171.1018 139.1117 211.1336	C_18_H_34_O_5_	3.76	Fatty acids	[77] CD
**44**	Trihydroxy-octadecadienoic acid (TriHODE) (*isomer I*)	25.48	327.2180	-	201.1126 213.1127 291.1967	C_18_H_32_O_5_	3.68	Fatty acids	CD
**45**	Trihydroxy-octadecadienoic acid (TriHODE) (*isomer II*)	26.11	327.2180	-	171.1019 201.1127 291.1961	C_18_H_32_O_5_	4.05	Fatty acids	CD
**46**	13-Oxo-octadecadienoic acid (13-Oxo-ODE)	26.61	-	295.2268	277.2162 179.1434	C_18_H_30_O_3_	0.95	Fatty acids	[77] CD
**47**	Dihydroxy octadecadienoic acid (DiHODE)	26.63	311.2230	-	293.2125 275.2019	C_18_H_32_O_4_	4.29	Fatty acids	[77] CD
**48**	Dihydroxy octadecadienoic acid (DiHODE) (*isomer I*)	26.76	311.2230	-	293.2125 275.2019	C_18_H_32_O_4_	4.69	Fatty acids	[77] CD
**49**	Dihydroxy octadecadienoic acid (DiHODE) (*isomer II*)	26.94	311.2231	-	293.2123 275.2020	C_18_H_32_O_4_	4.49	Fatty acids	[77] CD
**50**	13-Oxo-octadecadienoic acid (13-Oxo-ODE) (*isomer I*)	27.40	293.2126	-	275.2016 171.1017	C_18_H_30_O_3_	4.79	Fatty acids	[77] CD
**51**	*tris*-(Dihydrocaffeoyl) spermine	27.41	-	693.3461	293.0565	C_37_H_48_N_4_O_9_	−5.43	Hydroxycinnamic acid amides	[65,70]
**52**	Hydroxy-octadecadienoic acid (HODE)	27.86	295.2279	-	171.1018 277.2176	C_18_H_32_O_3_	4.05	Fatty acids	[77] CD
**53**	13-Oxo-octadecadienoic acid (13-Oxo-ODE) (*isomer II*)	28.24	293.2124	-	275.2015	C_18_H_30_O_3_	4.37	Fatty acids	[77] CD
**54**	Palmitic acid	28.91	255.2330	-	-	C_16_H_31_O_2_	4.07	Fatty acids	[72,74]
**55**	Linoleamide	29.36	-	280.2636	263.2368	C_18_H_33_NO	−0.34	Fatty acids	[80]
**56**	Palmitic amide	29.84	-	256.2636	88.0761 102.0913 172.1689	C_16_H_33_NO	0.47	Fatty acids	[80]
**57**	Oleamide	30.06	-	282.2789	265.2529 247.2418	C_18_H_36_NO	0.66	Fatty acids	[80] CD
**58**	Oleic acid	30.86	281.2487	-	-	C_18_H_34_O_2_	4.09	Fatty acids	[68] CD
**59**	Protocatechuate	31.01	153.0184	-	109.0283	C_7_H_5_O_4_^−^	0.89	Phenolic acids	[72]
**60**	Violaxanthin	34.24	-	601.4229	-	C_40_H_56_O_4_	−4.10	Xanthophylls	[81]

CD: compound discoverer. The reference column indicates a public/commercial spectral library and/or a literature study to confirm the putatively annotated compound.

**Table 2:** Quantitative analysis of the major compounds identified in LBE.

Peak	Compounds	µg CAE g^−1^ dw
**2**	*p*-Coumaric acid-hexoside (*isomer I*)	27.98 ± 1.38
**6**	3,4-di-*O*-caffeoylquinic acid	6.20 ± 0.18
**7**	Caffeic acid	1.07 ± 0.12
**8**	*p*-coumaric acid-hexoside (*isomer II*)	35.55 ± 2.84
**9**	3,5-di-*O*-caffeoylquinic acid	7.68 ± 1.98
**12**	Ferulic acid hexoside	<LOQ
**13**	*p*-coumaric acid-hexoside (*isomer III*)	<LOQ
**14**	*N^1^*-dihydrocaffeoyl, *N^10^*-caffeoyl spermidine	<LOQ
**16**	*p*-coumaric acid	214.43 ± 1.42
**19**	*p*-coumaroyl-quinic acid	2.38 ± 0.076
**23**	Ferulic acid	0.99 ± 0.18
**29**	*N-p-cis/trans*-coumaroyl-tyramine	<LOQ
**33**	*N-cis/trans*-feruloyltyramine	407.24 ± 20.86
**37**	*N-p-cis/trans*-coumaroyl-tyramine (*isomer I*)	53.05 ± 3.07
**39**	*N-cis/trans*-feruloyltyramine	522.52 ± 24.10
	Total Hydroxycinnamic Acids	1279.10 ± 0.05
		**µg RE g^−1^ dw**
**21**	Rutin hexose	18.49 ± 7.96
**26**	Rutin	1607.36 ± 89.85
**28**	Quercetin-3-*O*-hexoside	<LOQ
**30**	Naringenin-7-*O*-hexoside (Prunin)	1.15 ± 1.58
**31**	Kaempferol 3-*O*-hexoside-7-*O*-rhamnoside (Nictoflorin) (*isomer I*)	298.88 ± 17.93
**34**	Kaempferol-3-*O*-hexoside	<LOQ
**35**	Isorhamnetin-3-*O*-rutinoside	182.55 ± 36.32
**36**	Isorhamnetin-3-*O*-hexoside	<LOQ
**40**	Quercetin	<LOQ
**41**	Naringenin	14.91 ± 8.61
	Total Flavonoids	2123.34 ± 0.06
		**mg OAE g^−1^ dw**
**42**	Trihydroxy-octadecadienoic acid (TriHODE)	3.22 ± 0.30
**43**	Trihydroxy-octadecenoic acid	1201.76 ± 30.44
**44**	Trihydroxy-octadecadienoic acid (TriHODE) (*isomer I*)	276.61 ± 6.20
**45**	Trihydroxy-octadecadienoic acid (TriHODE) (*isomer II*)	440.45 ± 10.03
**47**	Dihydroxy octadecadienoic acid (DiHODE)	538.99 ± 6.96
**48**	Dihydroxy octadecadienoic acid (DiHODE) (*isomer I*)	435.75 ± 2.64
**49**	Dihydroxy octadecadienoic acid (DiHODE) (*isomer II*)	433.99 ± 7.60
**50**	13-Oxo-octadecadienoic acid (13-Oxo-ODE) (*isomer I*)	472.07 ± 18.54
**52**	Hydroxy-octadecadienoic acid (HODE)	1737.23 ± 23.17
**53**	13-Oxo-octadecadienoic acid (13-Oxo-ODE) (*isomer II*)	220.76 ± 9.00
**54**	Palmitic acid	<LOQ
**58**	Oleic acid	26.25 ± 0.33
	Total Fatty Acids	5787.08 ± 24.83

Data expressed as mean ± deviation standard (n = 3). CAE: caffeic acid equivalents; RE: rutin equivalents; OAE: oleic acid equivalents.

### 3.3. LBE Induces Pyroptosis in MCF-7 Cells, Saving Healthy Cells

The anticancer activity of LBE has initially been evaluated on MCF-7, MDA-MB-231, and SK-BR-3 human breast cancer cell lines. The control nontumorigenic breast cell line MCF-10A was used. Cell proliferation was examined by using the MTT assay. The viability of LBE-treated cells was measured after 24 h of treatment, using the extract in a concentration range of 3.125–100 µg/mL. The extract exhibited the strongest antiproliferative effects on the MCF-7 cell line (EC_50_ = 45 ± 3 μg/mL) compared to MDA-MB-231 (EC_50_ = 55 ± 2 μg/mL) and SK-BR-3 (EC_50_ = 60 ± 5 μg/mL).

**Figure 1:**
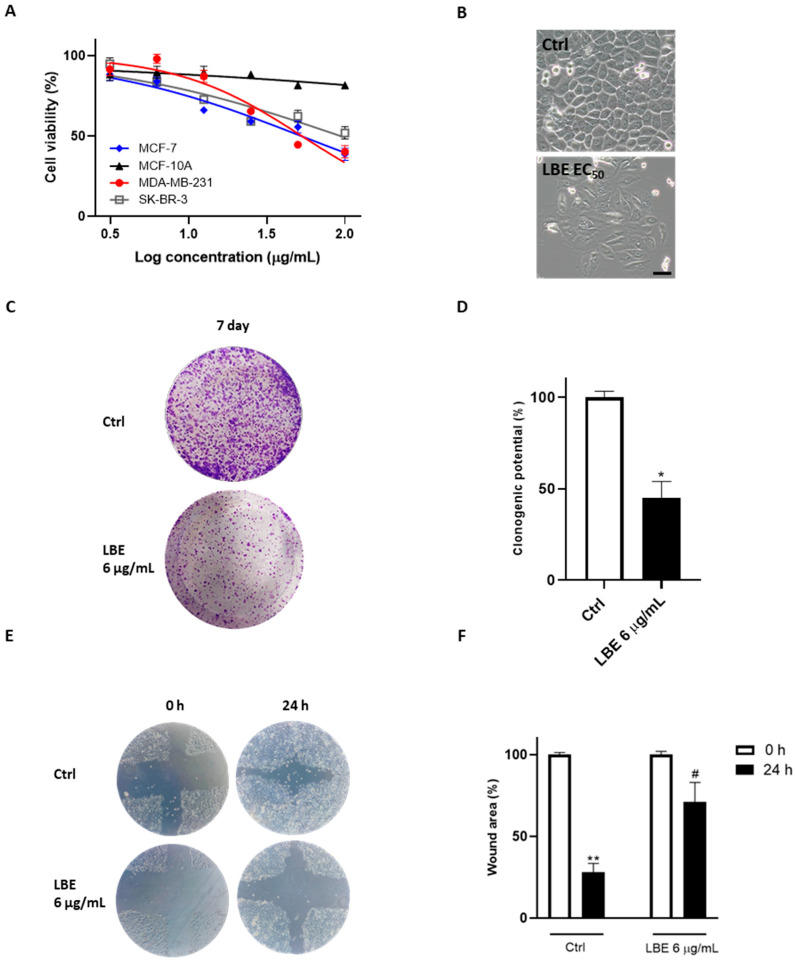
Cytotoxic effects of LBE on MCF-7 breast cancer cell line. Inhibitory action on cell proliferation of LBE was initially measured through MTT assay after 24 h of treatment of MCF-7, MDA-MB-231, and SK-BR-3 cell lines and MCF-10A healthy epithelial cell line (**A**). Then, EC_50_s were estimated. EC_50_ values are expressed as mean ± SD. (**B**) After 24 h of LBE exposure (EC_50_), morphological changes were observed using phase-contrast microscope, revealing pyroptotic features of MCF-7 cells treated with LBE. Magnification 40×. Scale bar: 50 μm. (**C**) Representative images of clonogenic assay in presence of LBE (6 μg/mL) for 7 days. (**D**) Histogram referring to optical density (OD) obtained from 1% SDS cell dissolution and measured by using spectrophotometer. (**E**) Effect of LBE exposure (6 μg/mL) on cell migration of MCF-7 cells through wound healing assay by using phase-contrast microscope. Magnification 20×. (**F**) Quantitative analysis of wound area of MCF-7 cells at time 0 and 24 h. Data are shown as mean ± SD of three different experiments performed in triplicate. * *p* < 0.05 vs. Ctrl; ** *p* < 0.01 vs. Ctrl; ^#^ *p* < 0.05 vs. Ctrl after 24 h.

Moreover, no toxicity was found on the MCF-10A healthy breast endothelial cell line (EC_50_ > 100 μg/mL), suggesting a selective mechanism of action against cancer cell lines (Figure 1A).

The observation of morphological changes using an inverted microscope revealed distinct swelling and membranolysis in MCF-7 cells after treatment with LBE (EC_50_) for 24 h (Figure 1B). Considering these findings, to acquire further insights into the cytotoxic effect showed by LBE, its clonogenic potential was evaluated on the MCF-7 cell line to assess its long-term interference in colony formation by individual cells. The results showed a reduction in colony formation after the administration of 6 μg/mL of LBE, with statistically significant differences compared to the control (LBE after 7 days: 47.58% clonogenic potential vs. Ctrl after 7 days; *p* < 0.05) (Figure 1C,D).

Subsequently, to examine the effect of LBE extract on tumor cell motility, a wound closure test was carried out on both untreated and LBE-treated MCF-7 cells. As can be seen in Figure 1E,F, a larger wound area after 24 h was observed in LBE-treated MCF-7 cells (69% wound area) compared to untreated cells (24% wound area).

**Figure 2:**
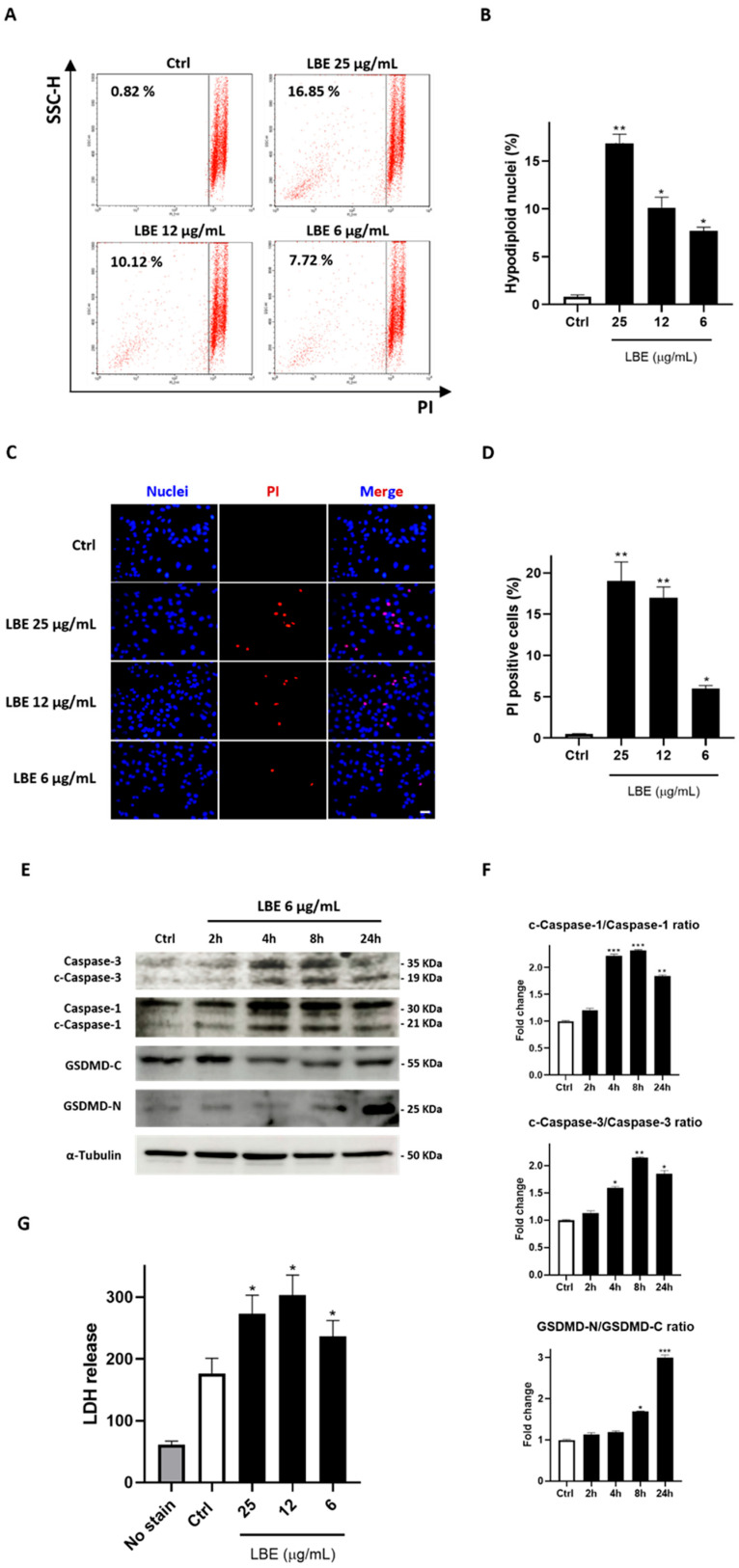
LBE selectively induces pyroptosis of MCF-7 breast cancer cell line. (**A**) After 24 h of LBE exposure (25, 12, 6 μg/mL), MCF-7 cells were stained with propidium iodide and fluorescence of hypodiploid nuclei (sub G0/G1) was measured by flow cytometry. (**B**) Quantitative analysis of hypodiploid nuclei was performed. (**C**) Hoechst 33342/PI double staining was performed to analyze dead and living cell distributions after exposure to LBE (25, 12, 6 μg/mL). (**D**) Quantitative analysis of PI-positive cells was performed (N ≥ 10). Scale bar: 20 μm. Cells were observed at 20× magnification. (**E**) Western blots of pyroptosis markers after exposure to LBE (6 μg/mL) at 2, 4, 8, and 24 h. (**F**) Densitometric analysis of Western blots. (**G**) LDH levels after exposure to LBE (25, 12, 6 μg/mL) at 24 h revealed by luminescence assay. Data are shown as mean ± SD of three different experiments performed in triplicate. * *p* < 0.05 vs. Ctrl; ** *p* < 0.01 vs. Ctrl; *** *p* < 0.001 vs. Ctrl.

To assess if the reduction in cell metabolization, observed through MTT assays, and the morphological changes were attributed to cell death induction, a propidium iodide (PI) staining test was performed. Flow cytometry analysis was then conducted to assess the formation of hypodiploid nuclei, which represent a hallmark of cell death. The results demonstrated a significant increase in the number of hypodiploid nuclei induced by LBE in a concentration-dependent manner (6–25 µg/mL) (Figure 2A,B). Interestingly, an accumulation of cellular debris resembling necrosis rather than an apoptosis event was observed, prompting a more detailed investigation into this phenomenon.

Thus, to investigate necrosis of MCF-7 due to LBE extract, PI/Hoechst 33342 double staining was carried out. Hoechst 33342 is a cell-permeable nuclear counterstain that emits blue fluorescence in combination with the minor groove of dsDNA. On the other hand, PI is a membrane-impermeable dye that labels dead cells which have lost their membrane integrity, emitting strong red fluorescence [82]. The results indicated that LBE significantly influenced necrotic events, consistent with the flow cytometry data (6–25 µg/mL; *p* < 0.01 vs. Ctrl at the highest concentration) (Figure 2C,D).

Therefore, based on the morphological analysis, we hypothesized that LBE treatment induces pyroptosis in breast cancer cells. This inference stems from the observation of cell permeability to PI, facilitated by the low molecular weight and pore formation associated with this regulated cell death form [83]. To confirm this hypothesis, Western blotting was used to measure the expression of pyroptosis markers. The results revealed the generation of cleaved caspase-1, cleaved caspase-3, and gasdermin D (GSDMD) and its N-terminal proteolytic fragment of GSDMD (GSDMD-N, active monomeric form), consistent with the activation of pyroptosis (Figure 2E,F) [84]. Caspase-1 causes the production of GSDMD-N, which is responsible for the formation of pores on the cell membrane. Following damage to the plasma membrane, lactate dehydrogenase (LDH) is released. In line with our results, LDH production was evaluated, revealing a significant increase in LDH levels following LBE administration compared to the control (6–25 µg/mL; *p* < 0.05 vs. Ctrl) (Figure 2G).

### 3.4. LBE Elicits Oxidative Stress in MCF-7 Cancer Cells and Exhibits Antioxidant Effects in Healthy Cells

Considering the potential pro-oxidant action of natural extracts on cancer cells and given that oxidative stress is a significant inducer of pyroptosis [85,86], intracellular ROS production following LBE treatment was evaluated. To this end, MCF-7 cells were treated with LBE (6 μg/mL) from 1 to 24 h, followed by the determination of ROS levels using DCFH-DA as the fluorescent agent. Doxorubicin (Doxo) was used as the positive control.

**Figure 3:**
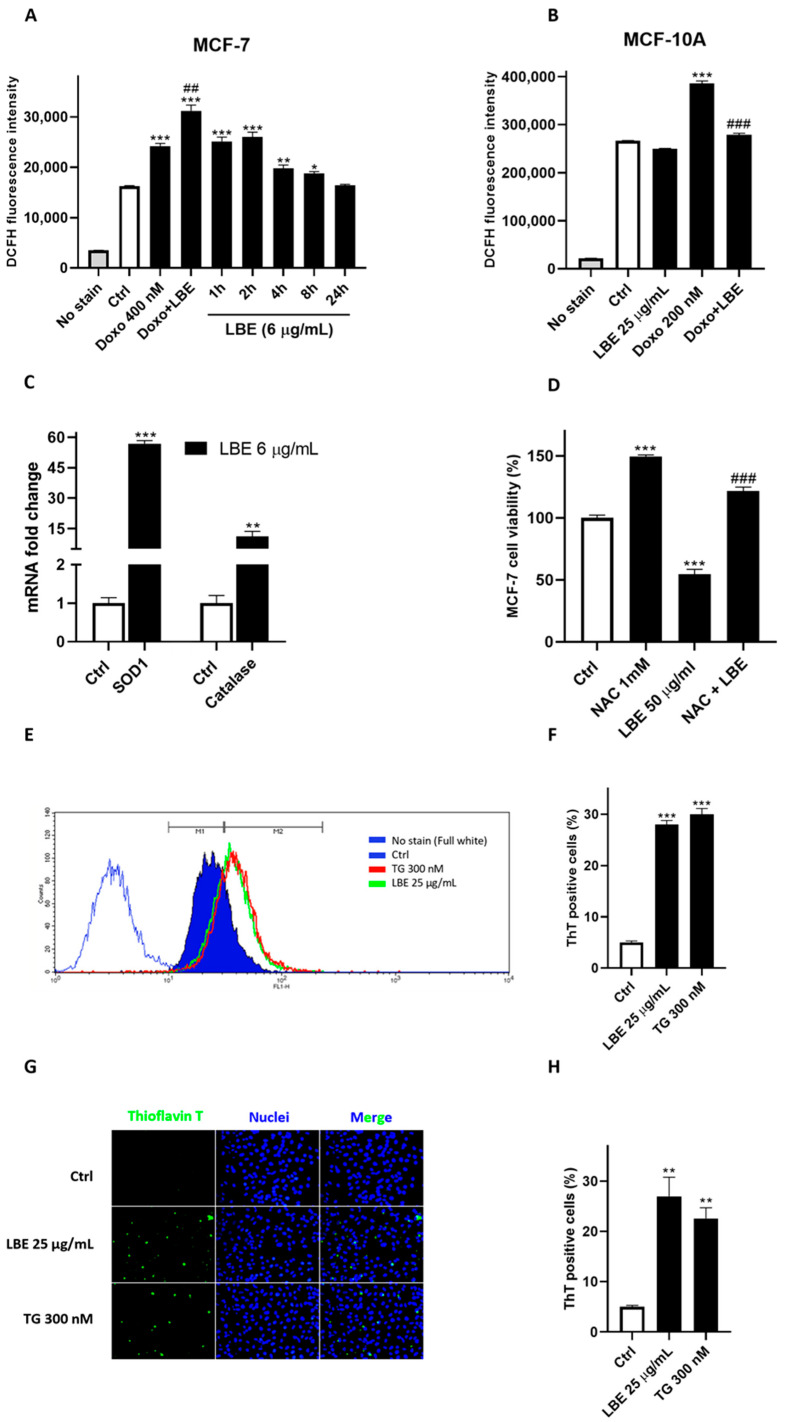
LBE selectively triggers oxidative stress in MCF-7 breast cancer cell line while protecting MCF-10A cell line from doxorubicin-induced ROS. 2′,7′-dichlorofluorescin diacetate (DCFH-DA) assay was conducted on MCF-7 (**A**) and MCF-10A cells (**B**) to reveal ROS production. Doxorubicin was used as positive control (400 and 200 nM for 4 h). Co-administration was assessed at 4 h. (**C**) Total RNA samples extracted from MCF-7 cells exposed for 24 h to LBE (6 μg/mL) were analyzed by q-PCR to detect CAT and SOD1 mRNA levels. GAPDH was used as housekeeping control. 2^−∆∆CT^ method was employed to calculate relative quantities of mRNA. Results are expressed as fold change relative to untreated cells. (**D**) MCF-7 cell viability using NAC as antioxidant corroborates pro-oxidant mechanism of LBE. ThT assay was performed for both flow cytometry (**E**) and fluorescence microscopy (**G**). Scale bar: 20 μm. (N ≥ 10). Cells were observed at 20× magnification. Thapsigargin 300 nM was used as positive control. (**F**,**H**) Quantitative analysis of ThT-positive cells was reported. Data are shown as mean ± SD of three different experiments performed in triplicate. * *p* < 0.05 vs. Ctrl; ** *p* < 0.01 vs. Ctrl; *** *p* < 0.001 vs. Ctrl. ^##^ *p* < 0.01 vs. doxorubicin; ^###^ *p* < 0.001 vs. doxorubicin.

As a result, a significant increase in ROS levels over a time range of 1–8 h was observed (Figure 3A), which may be indicative of an insult capable of triggering an inflammatory response induced by pyroptosis. Additionally, we detected possible synergism of LBE with Doxo, as their co-administration increased ROS compared to doxorubicin and LBE alone (LBE + Doxo vs. Doxo; *p* < 0.01; Figure 3A).

Furthermore, the antioxidant capacity of LBE was assessed, as previously determined in cell-free assays, to demonstrate its contemporary protective effects on the healthy epithelial cell line MCF-10A. At the same dosage that induced oxidative stress in the tumor line (6 μg/mL), we found no pro-oxidant/antioxidant effects. Meanwhile, at a concentration of 25 μg/mL, LBE significantly attenuated ROS production induced by the anticancer drug doxorubicin (LBE + Doxo vs. Doxo; *p* < 0.001; Figure 3B). Doxorubicin is known for its pro-oxidant effects at cardiac and endothelial levels, thereby limiting its therapeutic utility [87].

To corroborate the pro-oxidant activity of LBE on MCF-7 cells, the mRNAs levels of catalase and superoxide dismutase 1 (SOD1), key enzymes involved in cellular antioxidant defense mechanisms against various ROS, were quantified through q-PCR. As observed in Figure 3C, LBE leads to a substantial increase in mRNA production of these antioxidant enzymes after 24 h of treatment, acting as a possible stimulated protective response against oxidative stress induced by LBE treatment (6 μg/mL; *p* < 0.001 vs. Ctrl) in the time range of 1–8 h. Furthermore, to investigate whether the increased ROS level was responsible for LBE-induced cell death, cells were treated with the ROS scavenger NAC (N-acetyl cystein). As observed in Figure 3D, the results show that cell death caused by LBE was completely abrogated by NAC (*p* < 0.001 vs. LBE alone). These results suggest that the increased accumulation of intracellular ROS played an important role in LBE-induced cell death.

Therefore, LBE demonstrates a bifunctional capacity: inducing oxidative stress within cancer cells while simultaneously alleviating doxorubicin-induced oxidative stress in the healthy cellular environment.

Considering the delicate balance between oxidative stress and ER stress, the perturbation of ER homeostasis through the initiation of protein misfolding due to oxidative stress induced by LBE was evaluated. This phenomenon serves as a trigger for the UPR, which may potentially activate the inflammasome and lead to subsequent phenotypic manifestations, as demonstrated previously. Thioflavin T (ThT), a benzothiazole dye known for its enhanced fluorescence upon binding to protein aggregates, was employed as an indicator of UPR activation [88]. MCF-7 cells were cultured in the presence of LBE (25 μg/mL) or the ER stress-inducing agent, thapsigargin, serving as a positive control of UPR, for 24 h. Analyses were subsequently performed using both cytofluorimetry and fluorescence microscopy. Compared to the controls, the fluorescence significantly increased in LBE-treated cells, reaching levels comparable to those observed with thapsigargin (Figure 3E–H), thus representing, to our knowledge, the first demonstration of LBE impacting protein misfolding. Protein misfolding, unfortunately, was not detectable at 6 ug/mL, but we hypothesized that this was due to the weak brightness of the fluorophore, as observed in the flow cytometry and fluorescence microscopy images, which does not allow it to be as sensitive in detecting induced cellular misfolding at that low concentration. Instead, we hypothesized that by using higher concentrations (25 ug/mL), the accumulation of misfolded proteins is greater so it can be detected by thioflavin T.

### 3.5. LBE Induces ER Stress in MCF-7 Cancer Cells, Promoting Inflammasome Activation

Considering the activated UPR, a potential mechanism that links the induction of pyroptosis by ROS production with ER stress activation induced by the pro-oxidant effect of LBE was investigated.

Western blot analysis was performed to evaluate the increase in PKR-like ER kinase Ser/Thr protein kinase (PERK) expression, inositol-requiring Ser/Thr protein kinase 1α and RNA endonuclease (IRE1α) phosphorylation, as well as activating transcription factor 6 (ATF6) cleavage. Short times were specifically selected for the investigation, aligning with the functional timeframe of LBE concerning oxidative stress. This choice was also based on the role of the UPR, which typically aids cellular adaptation to such stress within a period of a few hours post-activation [9,89,90].

**Figure 4:**
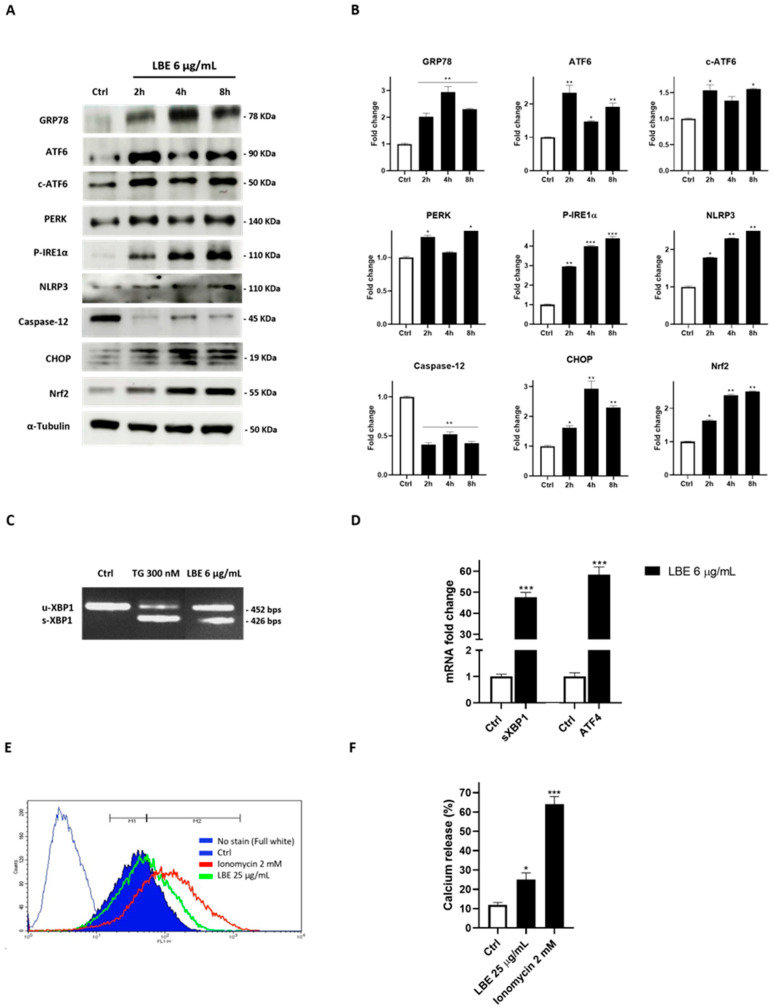
Biochemical effects of LBE on ER stress pathways. (**A**) Representative Western blot images showing increased expression of ER stress markers GRP78, ATF6, PERK, and P-IRE1α and some of their downstream targets (NLRP3, CHOP, Nrf2). Levels of cleaved proteins involved in UPR (c-caspase-12 and c-ATF6) were also evaluated. α-tubulin was used to check equal loading of protein extracts. Relative fold change vs. untreated cells, set as 1, is shown in graph. (**B**) Densitometric analysis of Western blots. (**C**) Total RNA samples extracted from MCF-7 cells exposed for 8 h to LBE were analyzed by RT-PCR to detect XBP1 mRNA splicing forms. (**D**) s-XBP1 and ATF4 mRNA ER stress hallmarks were assessed through q-PCR. GAPDH was used as housekeeping control. 2^–∆∆CT^ method was employed to calculate relative quantities of mRNA. Results are expressed as fold change relative to untreated cells. (**E**) Calcium mobilization analysis was performed with Mag fluo4-AM by flow cytometry. Ionomycin 2 mM was used as positive control. (**F**) Quantitative analysis of calcium-releasing cells was performed. Data are shown as mean ± SD of three different experiments performed in triplicate. * *p* < 0.05 vs. Ctrl; ** *p* < 0.01 vs. Ctrl; *** *p* < 0.001 vs. Ctrl.

Figure 4A shows that a concentration of 6 µg/mL of LBE led to a significant activation of all three transducers, indicative of a general cellular stress condition. As expected, the chaperone glucose regulatory protein 78 (GRP78), the master regulator of the UPR, was found to be overexpressed compared to the control. This overexpression is consistent with its role in regulating the binding release of the ER stress transducers, which also appear to be activated. Moreover, considering that the ER stress response ultimately leads to caspase-12 activation [91], its activation following LBE administration was evaluated. A notable reduction in its non-cleaved form was observed after LBE administration (Figure 4A,B). Considering the strong increase in p-IRE1α, the activation of its target NLPR3 inflammasome was determined. Additionally, considering the endoribonuclease activity of IRE1α, to confirm its activation, a semiquantitative PCR and q-PCR analysis was performed to evaluate the levels of unconventional mRNA splicing of X-Box Binding Protein 1 (XBP1), a specific transcription factor that undergoes excision of a 26-nucleotide unconventional intron from IRE1α [8]. As depicted in Figure 4C,D, we observed high levels of sXBP1 compared to the control (*p* < 0.001). The activation of PERK was also assessed through its downstream activation product, ATF4 (activating transcription factor 4), and we obtained significantly high levels of ATF4 compared to the control (*p* < 0.001) (Figure 4D). The remarkable activation of the ATF4 transcript, together with c-ATF6 and s-XBP1 activation, is fully compatible with the increased expression of their transcription factor CHOP (C/EBP Homologous Protein), as highlighted in Figure 4A. The CHOP protein, also known as GADD153 (growth arrest- and DNA damage-inducible gene 153), is activated as part of the UPR when ER stress is severe or prolonged. It plays a role in triggering pyroptosis through NLRP3 activation [92,93]. The expression of the antioxidant protein nuclear factor erythroid 2-related factor 2 (Nrf2), activated in a critical adaptive response to oxidative stress, was evaluated, revealing a time-dependent increase in its expression (Figure 4A). Nrf2 was also activated, inter alia, from PERK phosphorylation [94].

Elevated levels of ROS prompt the opening of the ER calcium channels IP3Rs and the ryanodine receptor, resulting in the augmented release of Ca^2+^ from the ER. This process further disrupts proper protein folding within the ER, further leading to NLRP3 inflammasome activation [95,96]. In this regard, the cytoplasmic calcium level was assessed. The effects are illustrated in a representative graph and the quantitative analysis result is depicted in Figure 4E,F, executed in a calcium-depleted buffer. Notably, a substantial alteration in Ca^2+^ concentrations was observed following LBE (25 μg/mL) administration, affirming its capacity to induce ER stress (LBE vs. Ctrl; *p* < 0.05). Ionomycin was employed as a positive control [97].

## 4. Discussion

Oxygen free radicals, ROS, and RNS play a dual role in biological systems. While they contribute to beneficial functions like bacterial destruction within phagocytic cells and programmed cell death, an imbalance favoring pro-oxidants, alongside reduced levels of antioxidant enzymes and endogenous antioxidants, can lead to an overproduction of ROS/RNS, resulting in oxidative stress. This redox imbalance is often linked to oncogenic stimulation and contributes to various aspects of tumor development and progression. Elevated ROS levels disrupt cellular processes by indiscriminately targeting proteins, lipids, and DNA [98,99]. Due to uncontrolled metabolic processes during hyperproliferation, adaptation to excessive ROS conditions in cancer cells suggests that they possess a higher basal level of antioxidative capacity and ROS than healthy cells.

Anticancer therapies that rely on oxidative damage aim to elevate the ROS level beyond the cytotoxic threshold to disrupt redox homeostasis and selectively eliminate cancer cells. Exogenous ROS-generating compounds make redox-imbalanced cancer cells more vulnerable than normal cells, resulting in cell death [100,101,102].

Therefore, in this study, we investigated the potential pro-oxidative action of LBE, aiming to utilize it as an anticancer agent to disrupt redox adaptation and induce ROS-dependent cytotoxicity in breast cancer cells. The results obtained in this study show that during the initial hours of treatment (1–8 h), LBE demonstrated a strong pro-oxidant action, resulting in a substantial increase in ROS concentration. This pronounced pro-oxidant action resulted in elevated levels of catalase and SOD1 mRNA at 24 h, consistent with previous findings in the literature [103,104]. The importance of the pro-oxidant mechanism was subsequently confirmed by using NAC as an antioxidant. Indeed, following its co-administration with LBE, the vitality of MCF-7 was fully restored.

Cancer cells exhibit elevated levels of transition metal ions, particularly copper and iron, which play pivotal roles in driving their rapid proliferation and metastasis. These metals serve as essential cofactors for various enzymes involved in critical cellular processes, such as DNA synthesis, cell signaling, and angiogenesis, facilitating the uncontrolled growth and spread of cancer cells. Moreover, given that numerous studies have documented the pro-oxidant activity of natural compounds in the presence of metal ions [105], we hypothesized that the elevated levels of ROS observed during the initial hours of treatment with LBE may also be attributed to the abundance of metal ions. In the presence of Cu^2+^, the pro-oxidant activity of phenolic compounds is expected to progress, leading to the generation of hydroxyl radicals (•OH). Meanwhile, excessive amounts of Fe^3+^ in a living cell can catalyze the generation of ROS via the Fenton reaction, which can damage DNA, lipids, proteins, and nucleic acids [106].

In recent years, ER stress has attracted widespread attention as a novel mechanism of cell death induced by natural compounds [107]. Oxidative stress induced by LBE disrupts ER homeostasis, resulting in the accumulation of misfolded or unfolded proteins. This accumulation triggers the UPR pathway, as evidenced by ThT staining. GRP78, recognized as the master regulator of the UPR, was found to be overexpressed relative to the control, indicating its role in regulating the binding release of ER stress transducers, which were also observed to be activated. Additionally, the extract significantly activated PERK expression, the phosphorylation of IRE1α, as well as ATF6 cleavage, all three being transducers compatible with a general cellular stress condition [108]. Furthermore, caspase-12, ATF4, sXBP1, and CHOP, downstream products of the ER stress response, were also highly activated after LBE treatment, demonstrating robust ER stress activation in cells. This phenomenon led to NLRP3 activation. The assembly of NLRP3 involves a multi-step process within cells and is associated with ER calcium release. Changes in cellular conditions, such as oxidative stress signals, can induce the release of calcium ions from the ER into the cytosol. This calcium release from the ER also serves as a signal in the process of NLRP3 inflammasome assembly and activation. Thus, we investigated its modulation following LBE administration, which increases its production as a signal of ER stress.

The activation of the NLRP3 inflammasome pathway leads to pyroptosis, a form of programmed cell death characterized by progressive cellular swelling until eventual membrane rupture. This rupture results in the release of cellular contents, triggering a robust inflammatory response. ROS-mediated pyroptosis induced by LBE is substantiated by the generation of cleaved caspase-1, cleaved caspase-3, GSDMD, and GSDMD-N, coupled with the release of LDH.

Although polyphenols have a significant impact on oxidative stress in cells, there are fewer studies demonstrating their antiproliferative efficacy against cancer cells compared to other components found in goji berries, such as polysaccharides. The precise mechanism of action remains unclear, and the specific components responsible for their anticancer activity have not been conclusively identified. Currently, the polyphenolic fraction of LBE has shown significant antioxidant effects and cytotoxicity against breast cancer cell lines, while the polysaccharide fraction contributes to its antiproliferative effects and ferroptosis induction [109,110]. A goji berry extract rich in polyphenols was tested on T-47D breast cancer cells, showing antitumor activity due to apoptosis via the mitochondrial pathway, indicated by a dose-dependent increase in pro-apoptotic Bax protein expression and a decrease in anti-apoptotic BclxL protein expression [111]. Aside from this study, to our knowledge, no other research has investigated the mechanism of toxicity of LBE polyphenols. Therefore, this investigation aims to extend these mechanistic aspects.

Moreover, there are no studies reporting the pharmacokinetics of the polyphenolic fraction of LBE. Identifying the potential mechanisms of action is crucial to understanding their pharmacokinetic properties and standardizing the concentration of these constituents. Indeed, a major limitation in using polyphenolic compounds is their bioavailability, which results in only a small fraction reaching the organs and tissues. However, while pharmacokinetic studies are necessary, existing research on polyphenol profiles similar to ours, as well as studies on the polysaccharides of LBE, suggests that the observed cellular concentrations may be compatible with the in vivo administration of LBE [112,113,114,115].

Furthermore, as evidence supporting the potential use of LBE in combination therapy for breast cancer, several combination tests were conducted with established drugs like doxorubicin. These tests revealed an augmentation in antitumor activity, alongside a potential dose-dependent decrease in the risk of cardiotoxicity associated with anthracycline-based therapeutic regimens for breast cancer [116,117,118].

## 5. Conclusions

Nutraceuticals have garnered significant attention in cancer research owing to their multifaceted effects and low toxicity profile. They play a crucial role in anticancer treatments by enhancing therapy efficacy, reducing drug concentrations to alleviate adverse effects, and delaying the development of therapy resistance. Despite their promising role in cancer treatment, the specific mechanisms underlying their actions remain largely unclear. This lack of mechanistic understanding can hinder their optimization and integration into clinical practice.

The present study aimed to conduct a comprehensive analysis of the onconutraceutical mechanism of the ethanolic extract derived from *Lycium barbarum* fruits. We demonstrated for the first time the inhibitory effects of LBE on cell proliferation through the induction of inflammatory programmed cell death in breast cancer cells. This effect is mediated by the pro-oxidant action of the extract, leading to the activation of ER stress. The results obtained showed that goji berry extract selectively triggers pyroptosis of tumor cells without having a significant toxic effect on healthy cells.

However, further studies are needed to elucidate the mechanism and the crucial role of ER stress in the activation of pyroptosis, as well as preclinical studies to corroborate these data. Furthermore, the current findings on the antitumor actions of LBE suggest that its bioactive compounds could be used as part of adjuvant therapeutic approaches in the potential treatment of cancer with anthracyclines, contributing to chemoprevention and tumor cell growth control.

Taken together, these findings highlight that *Lycium barbarum* may be a very promising natural extract in cancer therapy.

## Data Availability

Data are contained within the article and supplementary materials.

## Supplementary Materials

The following supporting information can be downloaded at: https://www.mdpi.com/article/10.3390/antiox13060708/s1, Figure S1: UV spectra (in range from 280 to 400 nm) of LBE (500 µg/mL) alone and in presence of 40 μM FeSO_4_, FeCl_3,_ and CuSO_4_. Figure S2. UHPLC-ESI(+)-Orbitrap-MS/MS spectra and proposed fragmentation pathway of *N^1^*-dihydrocaffeoyl, *N^10^*-caffeoyl spermidine hexose. Figure S3. UHPLC-ESI(+)-Orbitrap-MS/MS spectra and proposed fragmentation pathway of *N-p-trans*-coumaroyltyramine. Figure S4. UHPLC-ESI(−)-Orbitrap-MS/MS spectra and proposed fragmentation pathway of (a) caffeic acid, (b) p-coumaric acid, and (c) ferulic acid. Figure S5. UHPLC-ESI(−)-Orbitrap-MS/MS spectra and proposed fragmentation pathway of rutin. Figure S6. UHPLC-ESI(+)-Orbitrap-MS/MS spectra and proposed fragmentation pathway of kaempferol 3-O-hexoside-rhamnoside (nictoflorin). Figure S7. UHPLC-ESI(−)-Orbitrap-MS/MS spectra and proposed fragmentation pathway of hydroxy octadecadienoic acid (HODE). Figure S8. UHPLC-ESI(−)-Orbitrap-MS/MS spectra and proposed fragmentation pathway of trihydroxy octadecadienoic acid (TriHODE). Figure S9. UHPLC-ESI(+)-Orbitrap-MS/MS spectra and proposed fragmentation pathway of oleamide. Figures S10 and S11. Total ion chromatogram (TIC) profiles, in negative and positive ionization modes, respectively, of polyphenolic compounds isolated from *Lycium barbarum*. Figure S12. Western blots and PCR raw data. Table S1: Method validation parameters for quantitative assay.
