# Supplementary material for: Anticancer Therapies Based on Oxidative Damage: Lycium barbarum Inhibits the Proliferation of MCF-7 Cells by Activating Pyroptosis through Endoplasmic Reticulum Stress
Source: Antioxidants (Basel). 2024 Jun 11;13(6):708. doi: 10.3390/antiox13060708 (PMC11200455; doi:10.3390/antiox13060708)
Supplement: Supplementary file 1 [file antioxidants-13-00708-s001.zip › antioxidants-3014772-supplementary.pdf]

Supplementary Materials

Anticancer therapies based on oxidative damage: *Lycium barbarum* inhibits the proliferation of breast cancer cells by activating pyroptosis via endoplasmic reticulum stress

Maria Rosaria Miranda [1,2,†], Manuela Giovanna Basilicata [3,†], Vincenzo Vestuto [1,*], Giovanna Aquino [1,2], Pasquale Marino [4], Emanuela Salviati [1], Tania Ciaglia [1], Gloria Domínguez-Rodríguez [5], Ornella Moltedo [2], Luigi Coppola [6], Pietro Campiglia [1], Giacomo Pepe [1,7,*] and Michele Manfra [4]

[1] Department of Pharmacy, University of Salerno, Via G. Paolo II, Fisciano, 84084 Salerno, Italy; mmiranda@unisa.it; vvestuto@unisa.it; gaquino@unisa.it; esalviati@unisa.it; tciaglia@unisa.it; moltedo@unisa.it; pcampiglia@unisa.it

[2] PhD Program in Drug Discovery and Development, University of Salerno, Fisciano, 84084 Salerno, Italy.

[3] Department of Advanced Medical and Surgical Sciences, University of Campania "Luigi Vanvitelli", Naples, Italy; manuelagiovanna.basilicata@unicampania.it

[4] Department of Science, University of Basilicata, Viale dell'Ateneo Lucano 10, 85100 Potenza, Italy; pasqualemarino.pm90@gmail.com; michele.manfra@unibas.it

[5] Universidad de Alcalá, Departamento de Química Analítica, Química Física e Ingeniería Química, Facultad de Ciencias, Ctra. Madrid-Barcelona Km. 33.600, 28871 Alcalá de Henares, Madrid, Spain; gloria.dominguezr@uah.es

[6] DO.DA.CO. s.r.l., via delle Industrie, traversa Flavio Gioia, 84018 Salerno, Italy; luigi.coppola@dodaco.eu

[7] NBFC, National Biodiversity Future Center, 90133 Palermo, Italy; gipepe@unisa.it

* Correspondence: vvestuto@unisa.it; gipepe@unisa.it

† These authors are co-first authors

Figure S1. UV spectra (in range 280 to 400 nm) of LBE (500 µg/mL) alone and in the presence of 40 µM $FeSO_4$, $FeCl_3$ and $CuSO_4$.

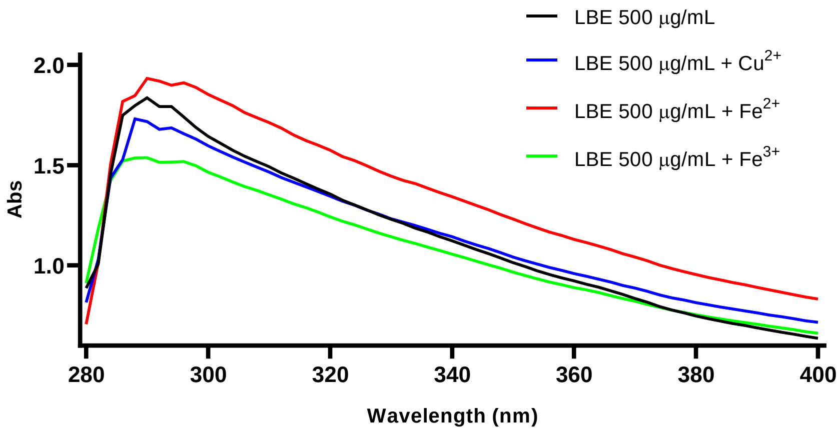

Figure S2. UHPLC-ESI(+)-Orbitrap-MS/MS spectra and proposed fragmentation pathway of *N*[1]-Dihydrocaffeoyl, *N*[10]-caffeoyl spermidine hexose.

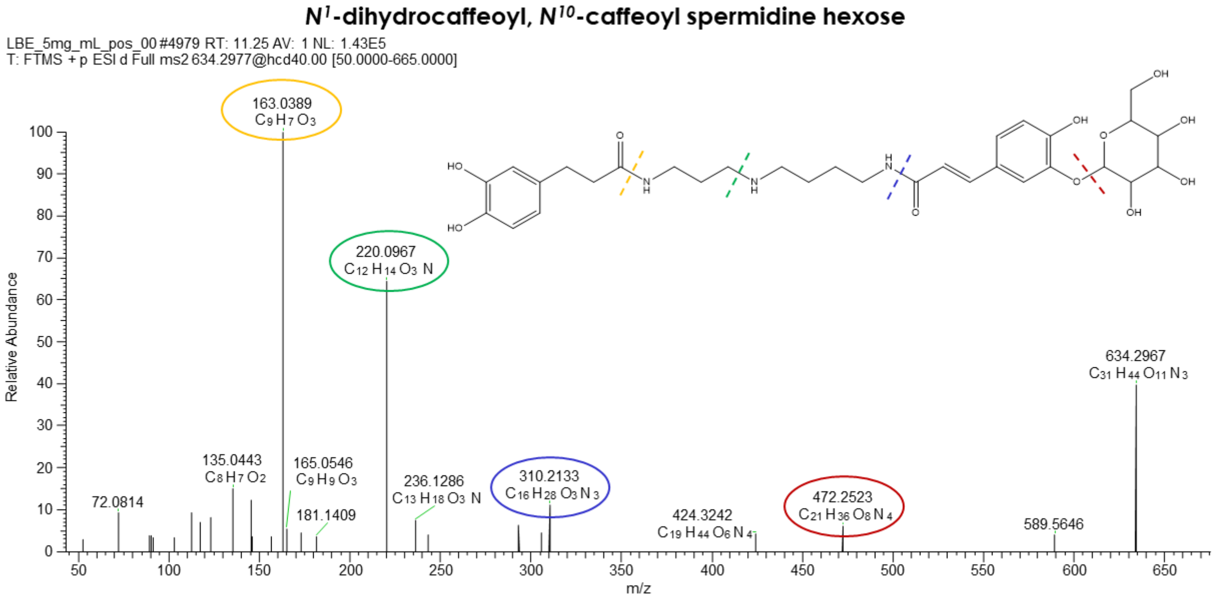

Figure S3. UHPLC-ESI(+)-Orbitrap-MS/MS spectra and proposed fragmentation pathway of *N-p-trans*-coumaroyltyramine.

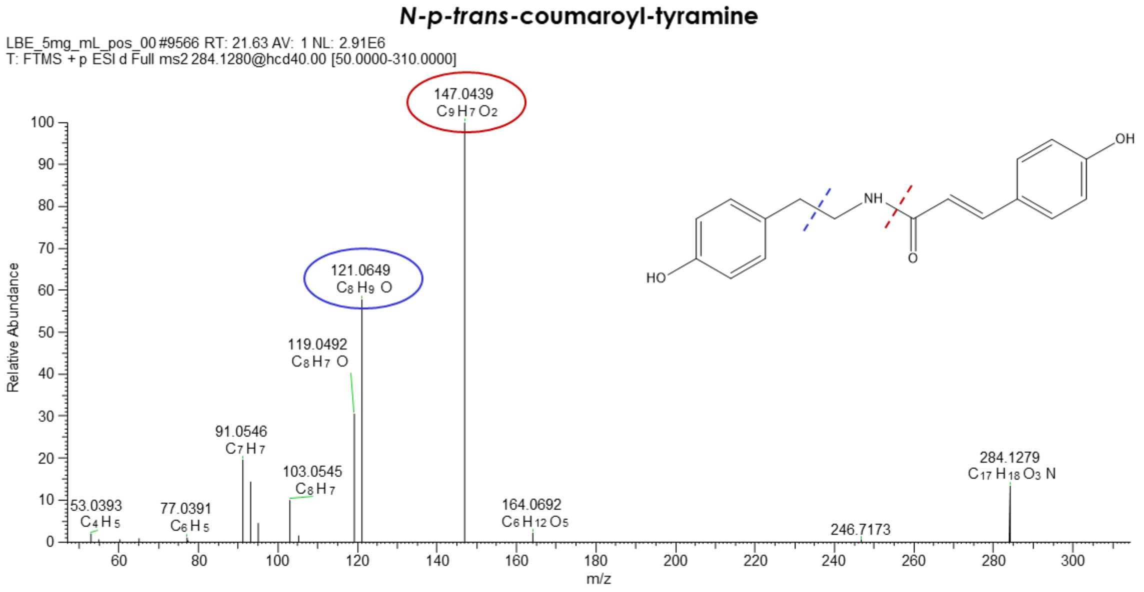

Figure S4. UHPLC-ESI(–)-Orbitrap-MS/MS spectra and proposed fragmentation pathway of a) Caffeic acid, b) p-coumaric acid, c) ferulic acid.

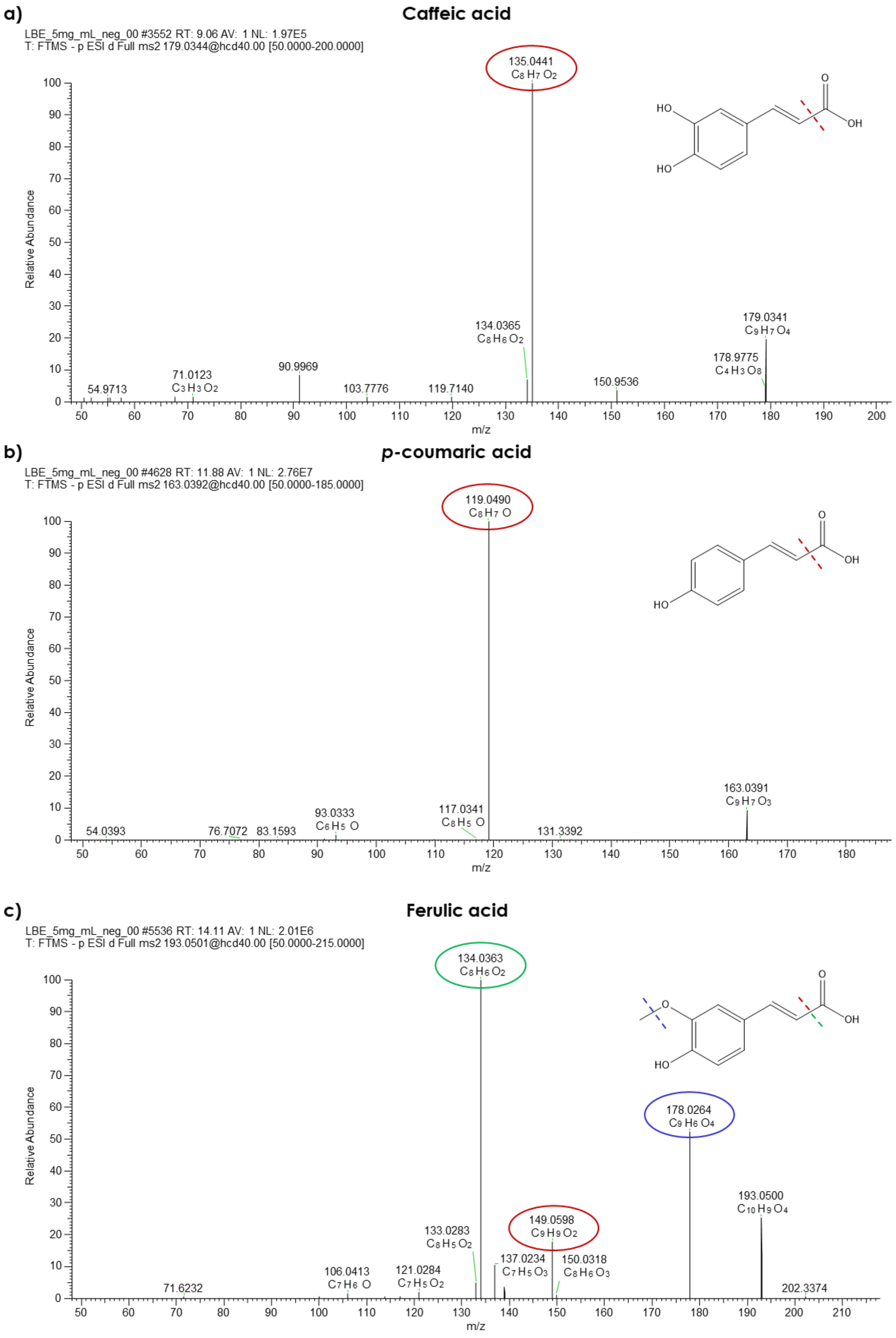

Figure S5. UHPLC-ESI(–)-Orbitrap-MS/MS spectra and proposed fragmentation pathway of Rutin.

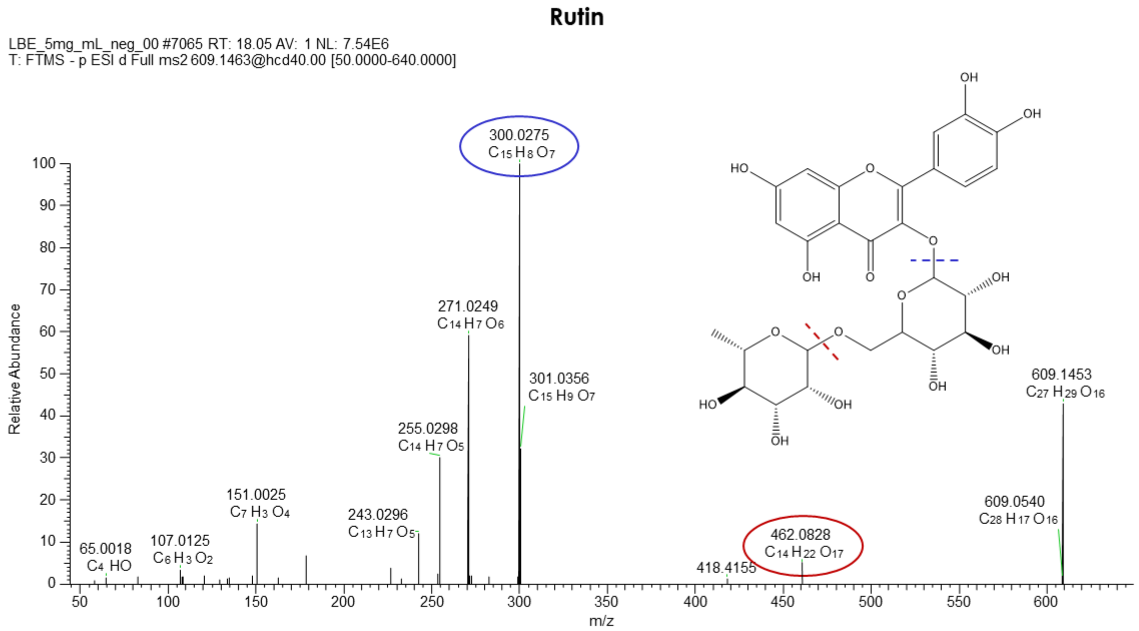

Figure S6. UHPLC-ESI(+)-Orbitrap-MS/MS spectra and proposed fragmentation pathway of Kaempferol 3-O-hexoside-rhamnoside (Nictoflorin).

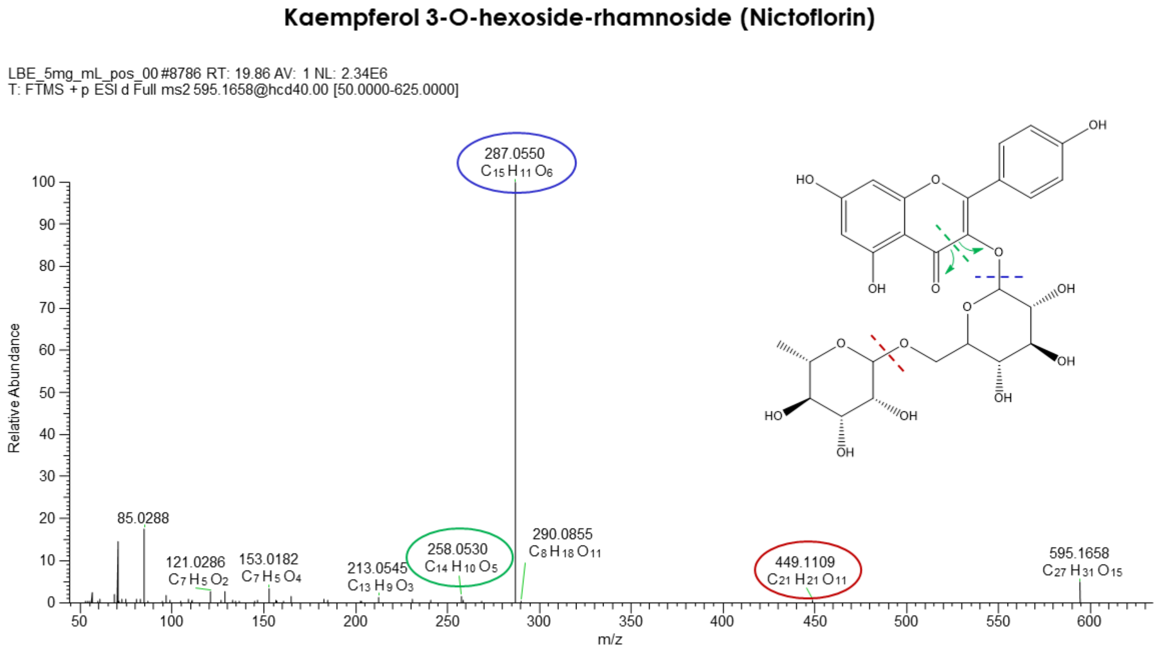

Figure S7. UHPLC-ESI(–)-Orbitrap-MS/MS spectra and proposed fragmentation pathway of Hydroxy octadecadienoic acid (HODE).

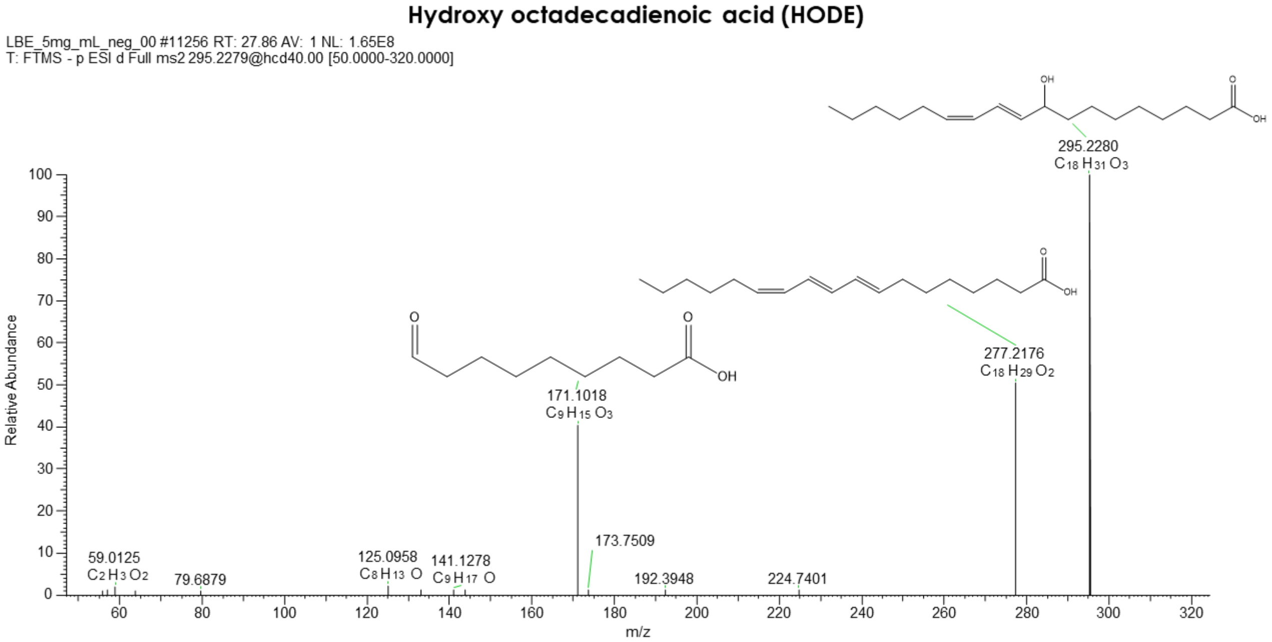

Figure S8. UHPLC-ESI(–)-Orbitrap-MS/MS spectra and proposed fragmentation pathway of Trihydroxy octadecadienoic acid (TriHODE).

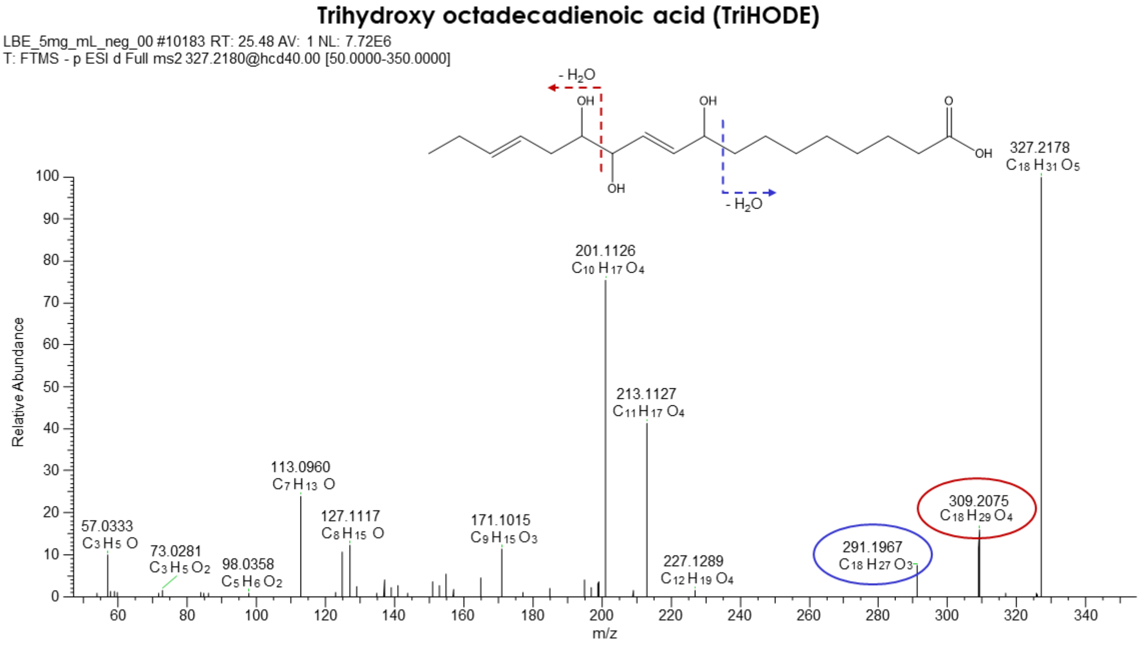

Figure S9. UHPLC-ESI(+)-Orbitrap-MS/MS spectra and proposed fragmentation pathway of Oleamide.

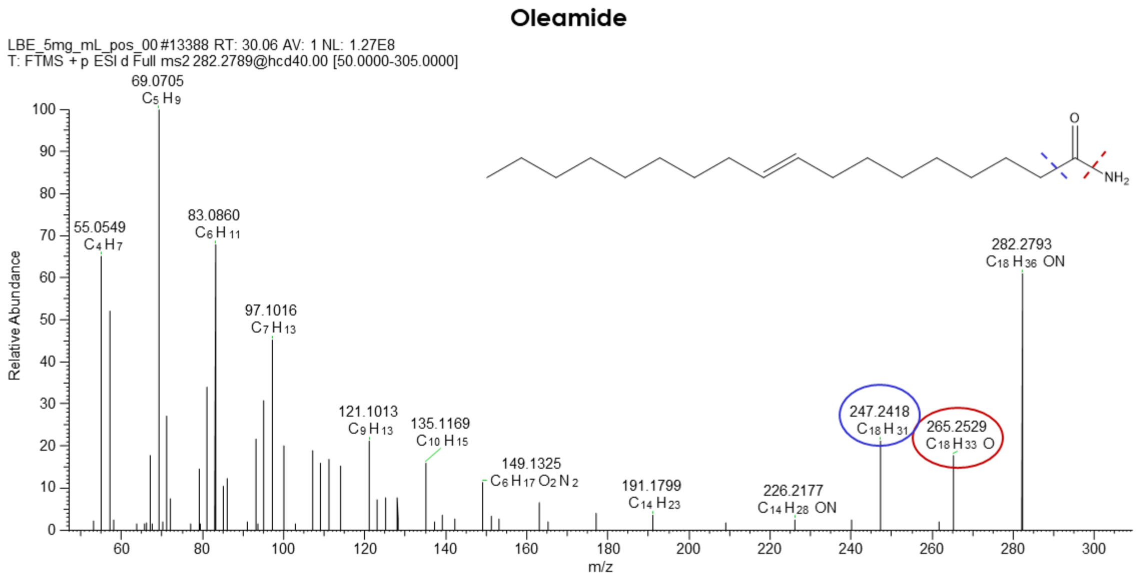

Figure S10 and S11. Total Ion Chromatogram (TIC) profiles, in negative and positive ionization modes, respectively, of polyphenolic compounds isolated from *Lycium barbarum*.

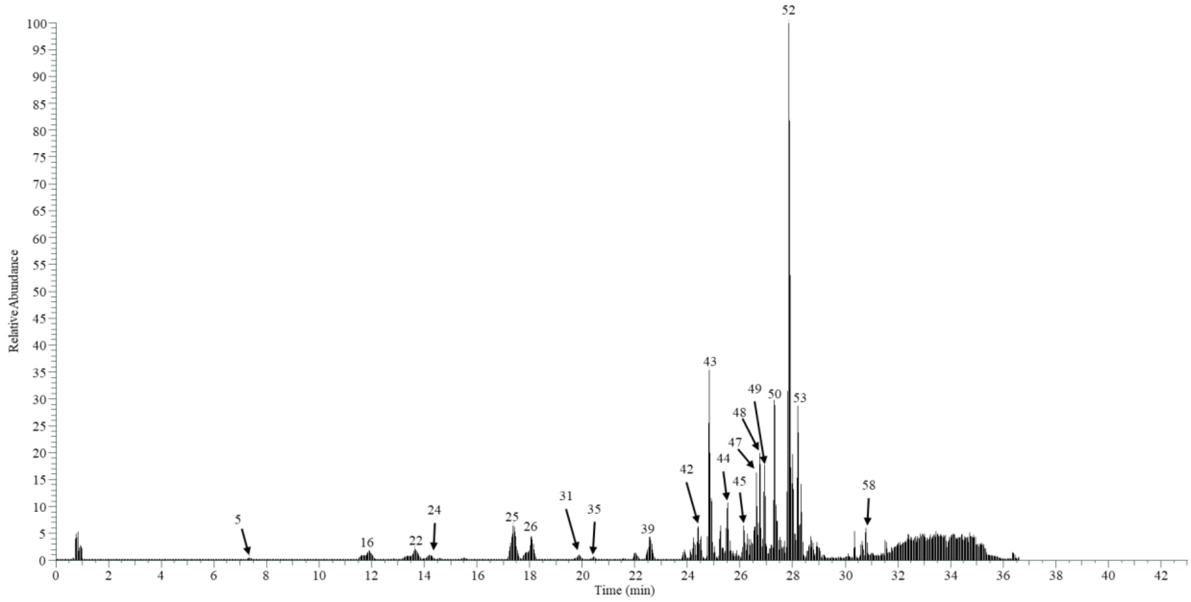

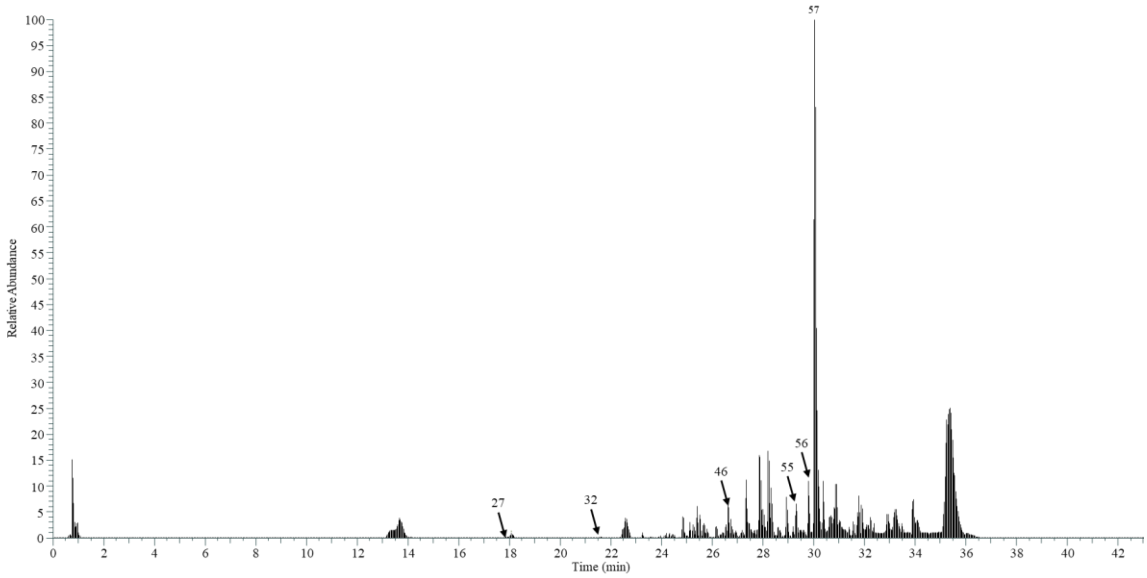

Figure S12. Western blots and PCR raw data

WB1: ATF6/P-IRE1/α-TUBULIN

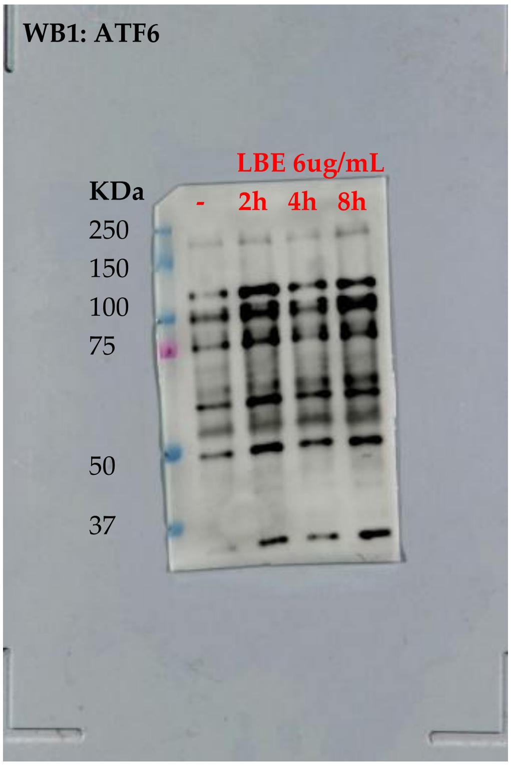

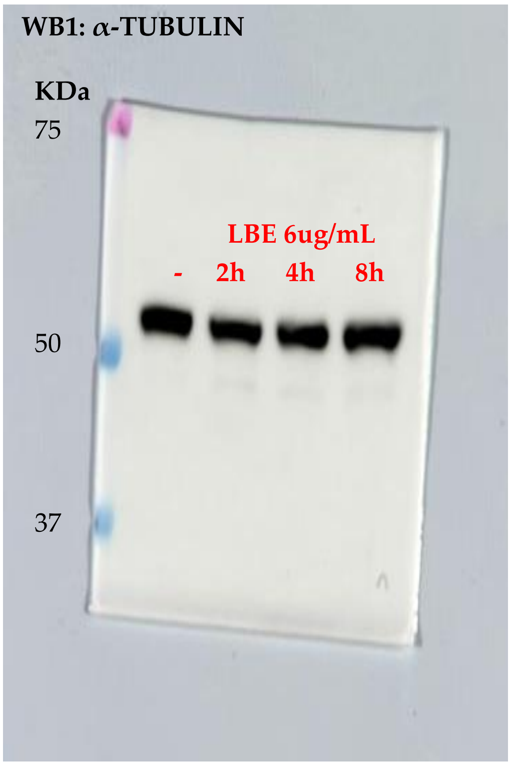

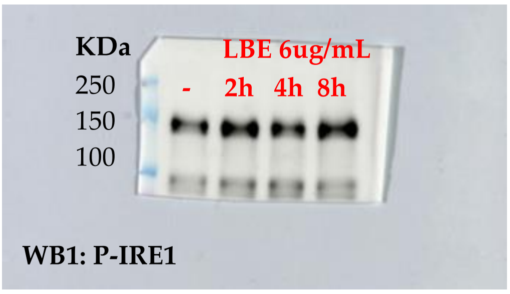

WB2: CASPASE-12/α-TUBULIN

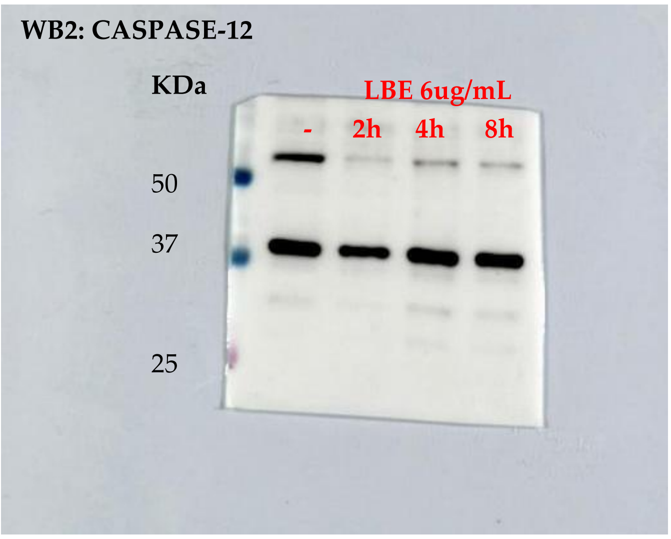

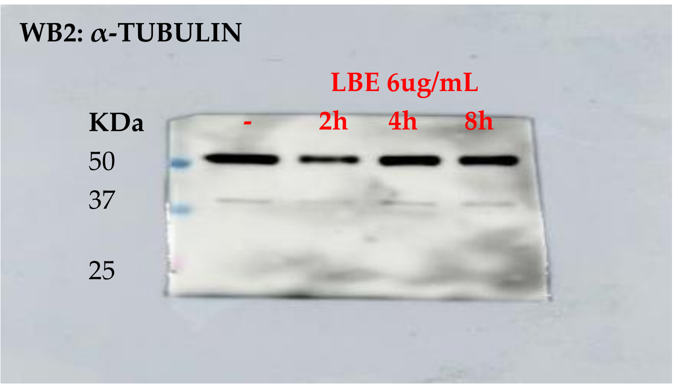

WB3: GRP78/NRF2/P-IRE1/α-TUBULIN

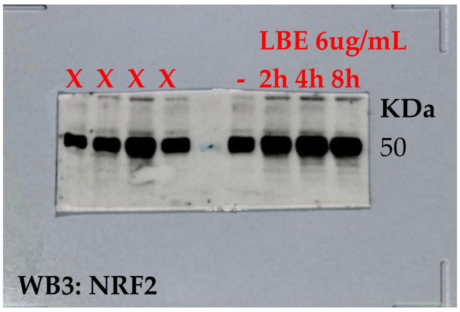

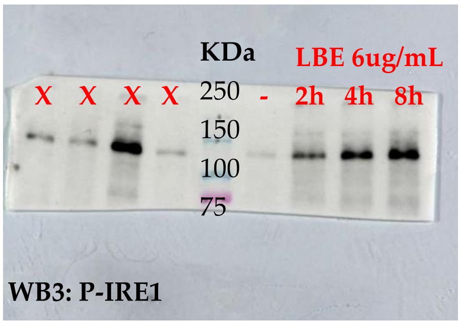

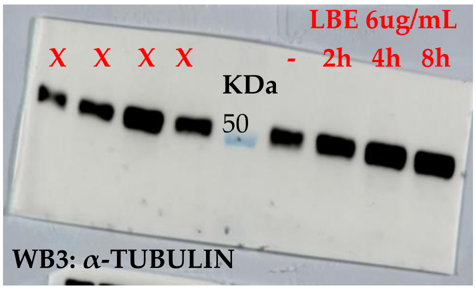

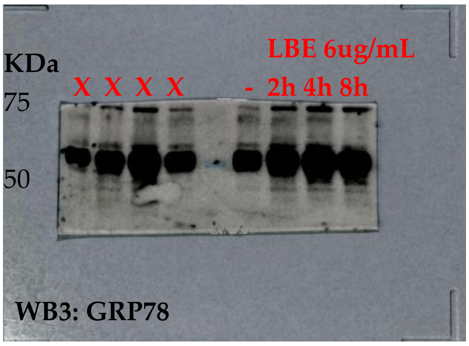

WB4: CASPASE-1/CASPASE-3/α-TUBULIN

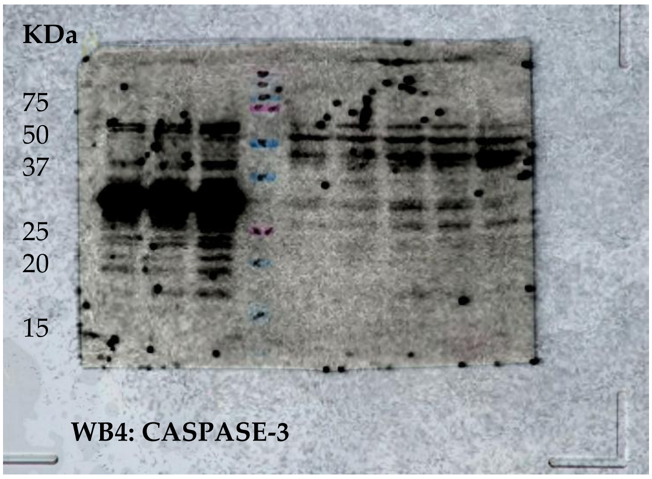

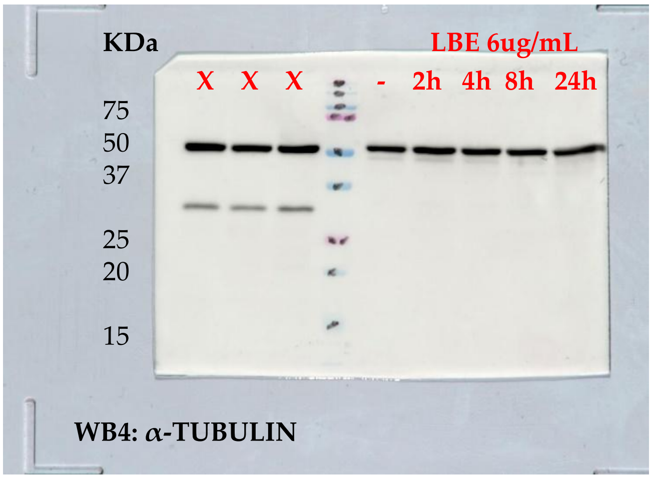

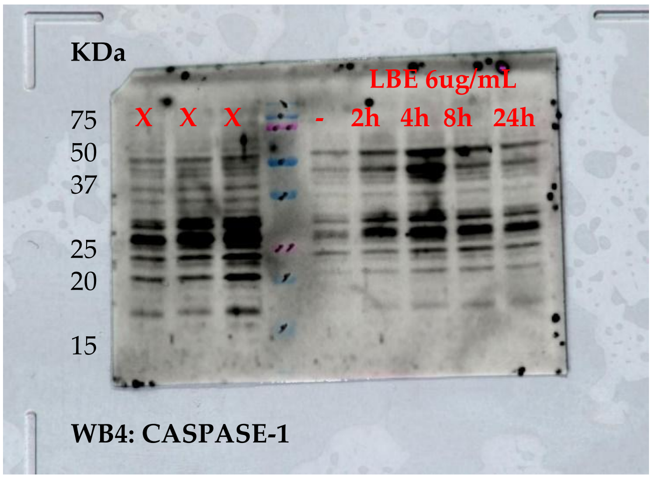

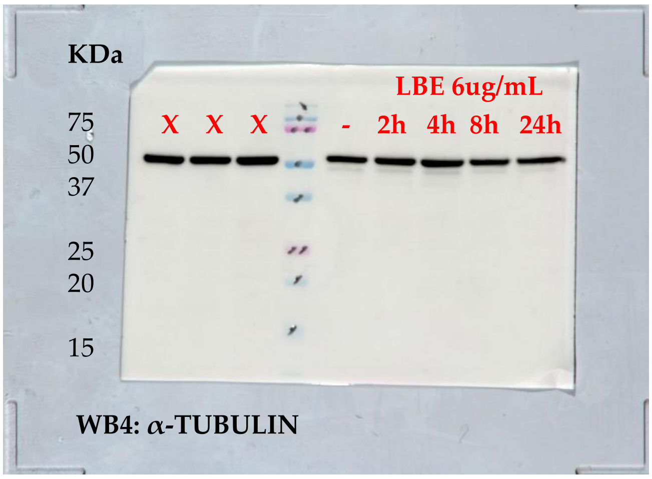

WB5: GSDMD/GAPDH

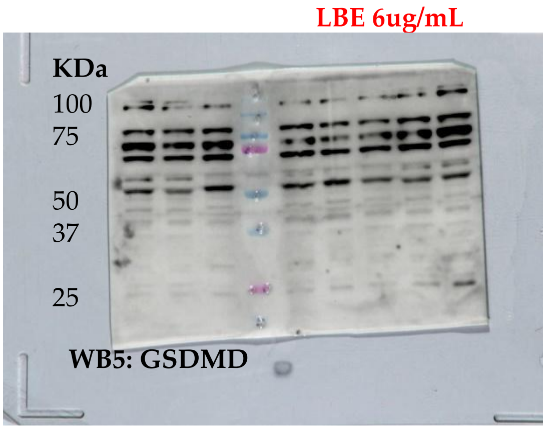

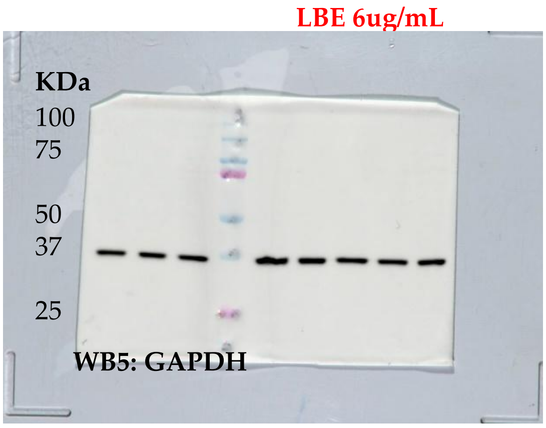

WB6: NLRP3/α-TUBULIN

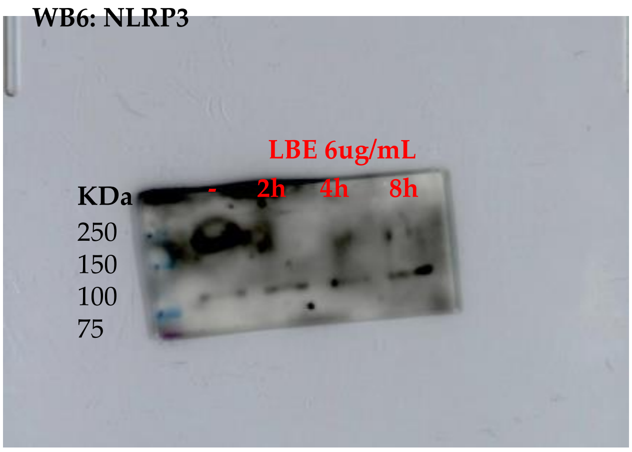

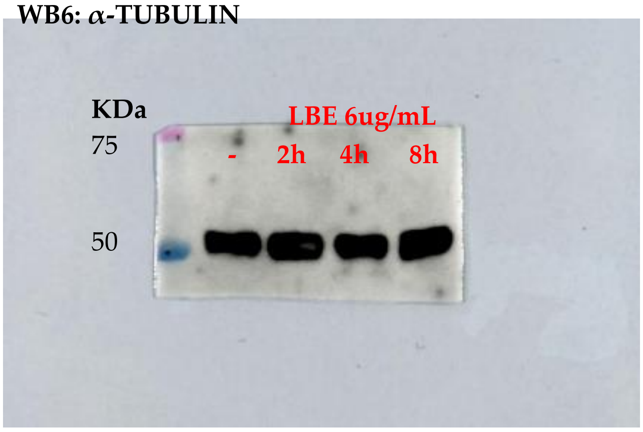

WB7: CHOP/α-TUBULIN

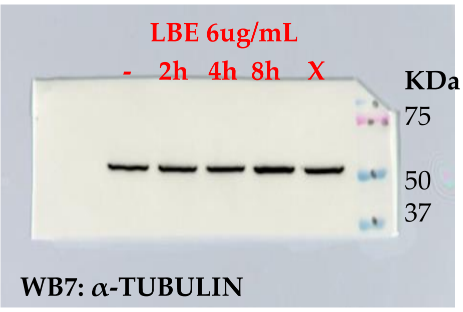

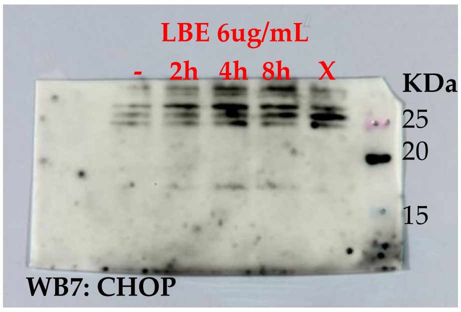

Xbp1 SPLICING

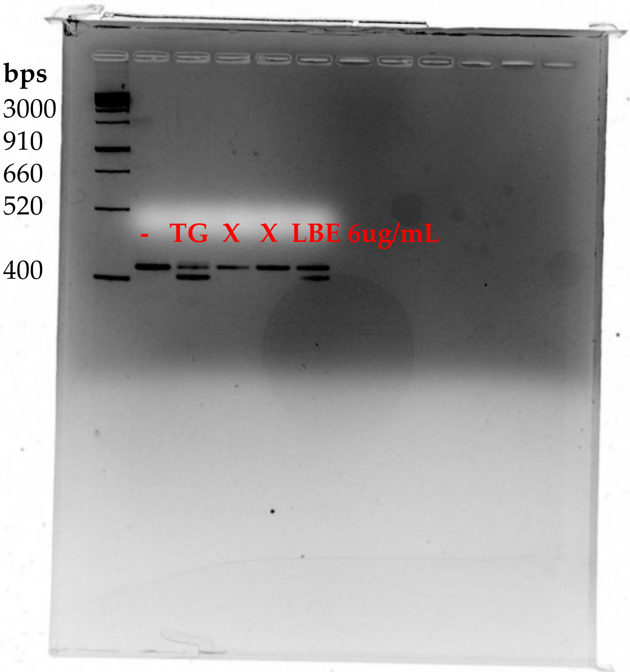

Figure S13. Microscopy images raw data

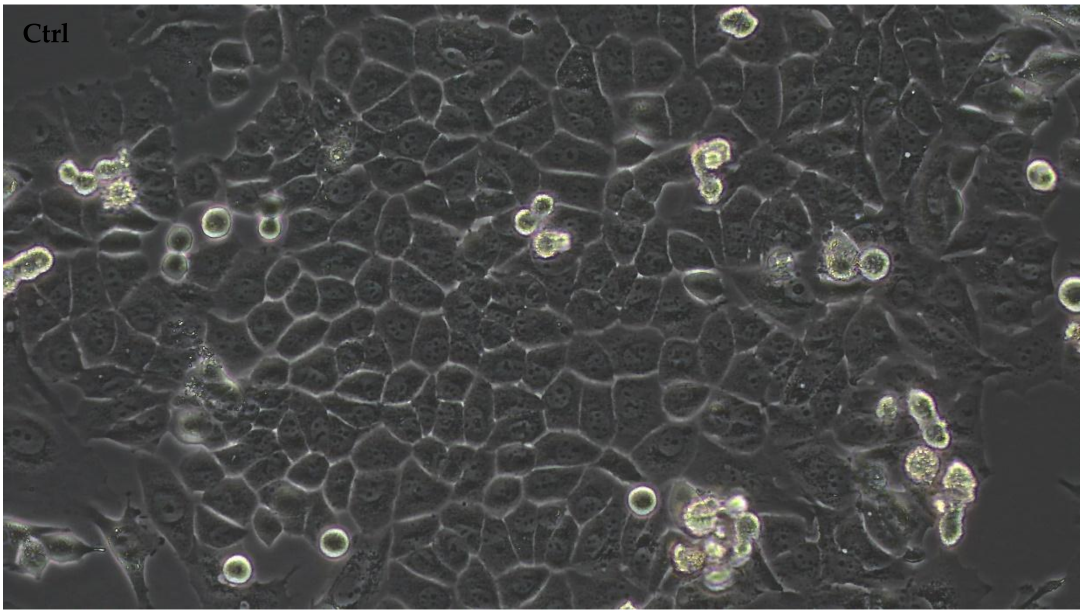

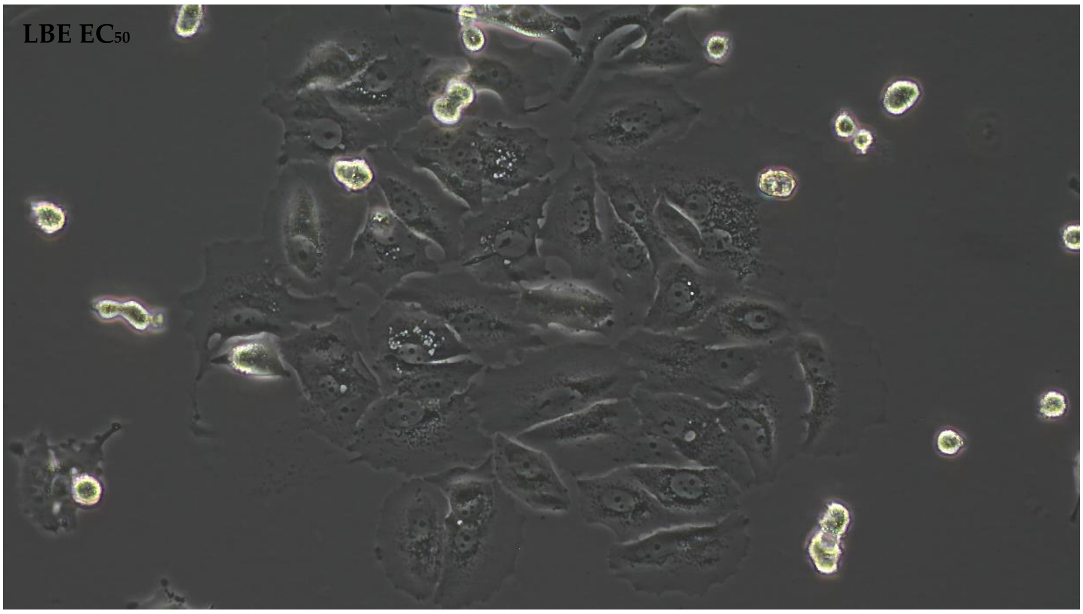

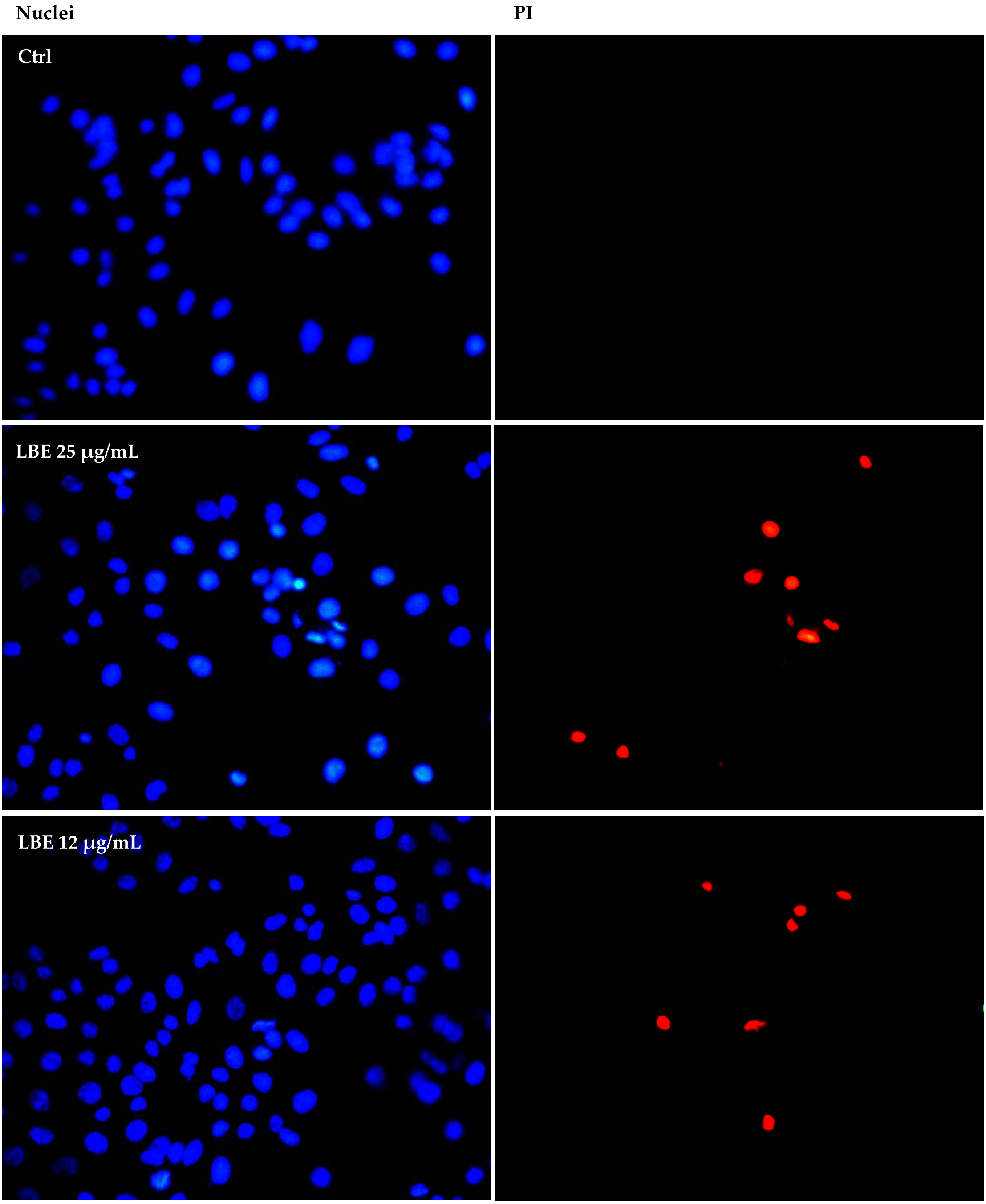
Nuclei
PI
Ctrl
LBE 25 μg/mL
LBE 12 μg/mL

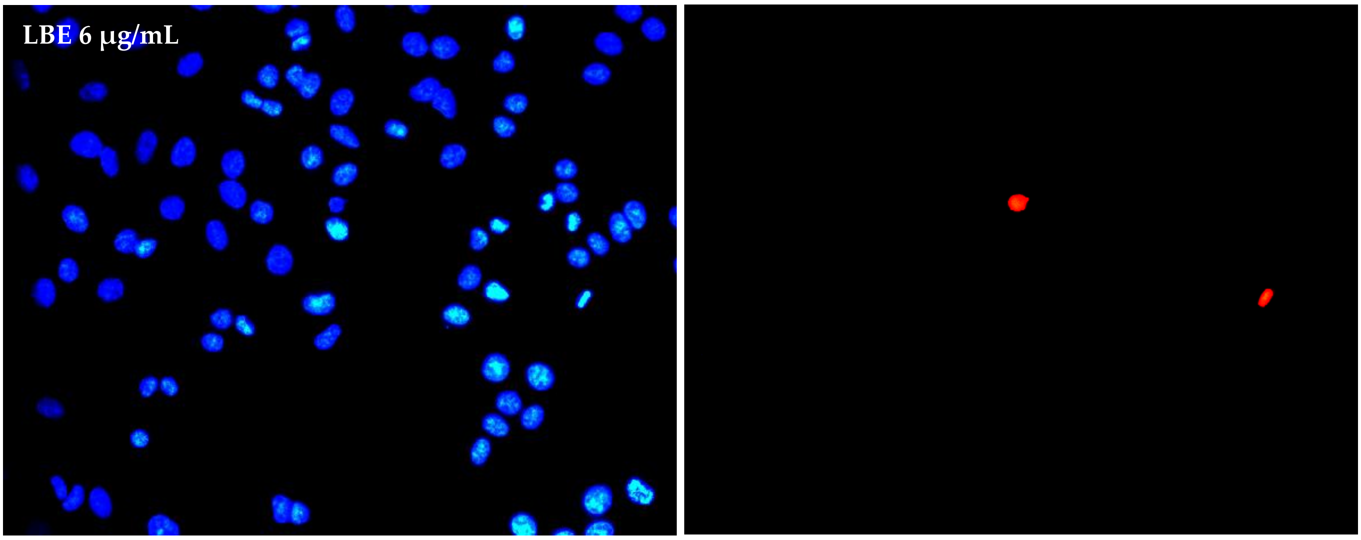
LBE 6 µg/mL

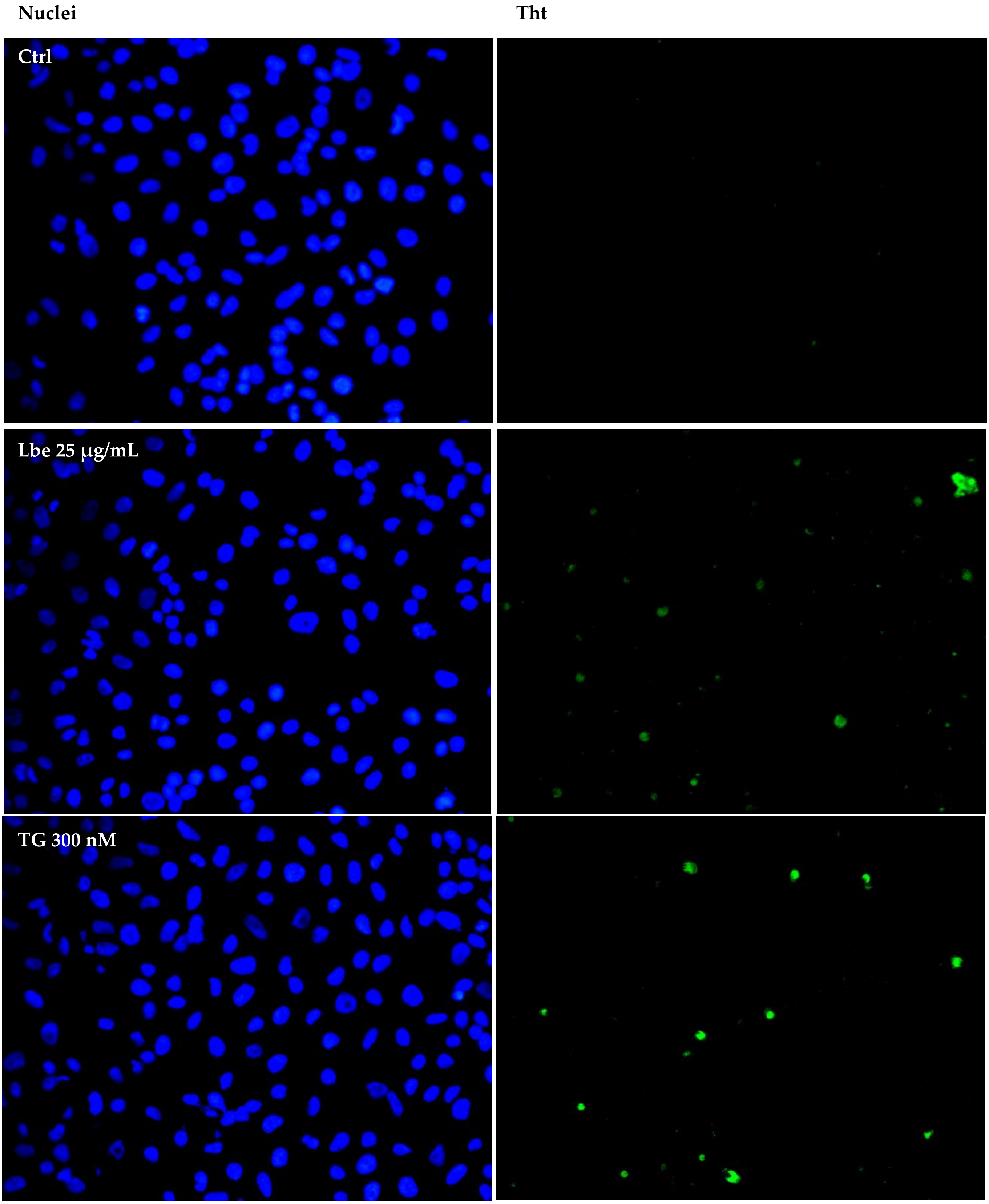
Nuclei
Tht
Ctrl
Lbe 25 µg/mL
TG 300 nM

Table S1. Method validation parameters for quantitative assay.

Parameters	Caffeic acid	Rutin	Oleic acid
**Retention time (min) ± dev. st. (n = 4)**	8.9425 ± 0.0096	17.1625 ± 0.0096	33.6850 ± 0.0058
**Regression equation**	y = 3.63E-09x -4.72E-02	y = 7.31E-09x -1.68E-01	y = 1.73E-06x -1.50E+01
**Linear range**	0.03-4 μg mL^{-1}	0.06-15.63 μg mL^{-1}	15.63-2000 μg mL^{-1}
**Correlation coefficient (R^2)**	≥ 0.9979	≥ 0.9989	≥ 0.9996
**Intraday (n = 3) Analyte concentration (μg mL^{-1}): Accuracy (%)**	0.98 : 91.68<br>1.95 : 95.63<br>3.91 : 101.91	1.95 : 90.77<br>3.91 : 91.35<br>7.81 : 100.31	62.5 : 92.19<br>125 : 93.34<br>250 : 95.03
**Interday (n = 3) Analyte concentration (μg mL^{-1}): Accuracy (%)**	0.98 : 90.42<br>1.95 : 94.81<br>3.91 : 102.41	1.95 : 105.57<br>3.91 : 100.76<br>7.81 : 104.48	62.5 : 97.48<br>125 : 109.11<br>250 : 92.28
**LOQ (μg mL^{-1})**	0.0008	0.0053	9.3185
**LOD (μg mL^{-1})**	0.0003	0.0017	3.0751
